# Predictive Models May Complement or Provide an Alternative to Existing Strategies for Assessing the Enteric Pathogen Contamination Status of Northeastern Streams Used to Provide Water for Produce Production

**DOI:** 10.3389/fsufs.2020.561517

**Published:** 2020-10-06

**Authors:** Daniel L. Weller, Tanzy M. T. Love, Alexandra Belias, Martin Wiedmann

**Affiliations:** 1 Department of Food Science, Cornell University, Ithaca, NY, United States; 2 Department of Biostatistics and Computational Biology, University of Rochester, Rochester, NY, United States

**Keywords:** agricultural water, *stx*, *eaeA*, *Salmonella*, *E. coli*, machine learning, predictive model

## Abstract

While the Food Safety Modernization Act established standards for the use of surface water for produce production, water quality is known to vary over space and time. Targeted approaches for identifying hazards in water that account for this variation may improve growers’ ability to address pre-harvest food safety risks. Models that utilize publicly-available data (e.g., land-use, real-time weather) may be useful for developing these approaches. The objective of this study was to use pre-existing datasets collected in 2017 (*N* = 181 samples) and 2018 (*N* = 191 samples) to train and test models that predict the likelihood of detecting *Salmonella* and pathogenic *E. coli* markers (*eaeA, stx*) in agricultural water. Four types of features were used to train the models: microbial, physicochemical, spatial and weather. “Full models” were built using all four features types, while “nested models” were built using between one and three types. Twenty learners were used to develop separate full models for each pathogen. Separately, to assess information gain associated with using different feature types, six learners were randomly selected and used to develop nine, nested models each. Performance measures for each model were then calculated and compared against baseline models where *E. coli* concentration was the sole covariate. In the methods, we outline the advantages and disadvantages of each learner. Overall, full models built using ensemble (e.g., Node Harvest) and “black-box” (e.g., SVMs) learners out-performed full models built using more interpretable learners (e.g., tree- and rule-based learners) for both outcomes. However, nested *eaeA-stx* models built using interpretable learners and microbial data performed almost as well as these full models. While none of the nested *Salmonella* models performed as well as the full models, nested models built using spatial data consistently out-performed models that excluded spatial data. These findings demonstrate that machine learning approaches can be used to predict when and where pathogens are likely to be present in agricultural water. This study serves as a proof-of-concept that can be built upon once larger datasets become available and provides guidance on the learner-data combinations that should be the foci of future efforts (e.g., tree-based microbial models for pathogenic *E. coli*).

## INTRODUCTION

The occurrence of multiple foodborne disease outbreaks, over the past two decades, that were potentially linked to the use of contaminated water to irrigate fresh produce has increased concerns over the use of surface water for produce production (Ackers et al., 1998; Centers for Disease Control and Prevention, 2008; Greene et al., 2008; Söderström et al., 2008; Food and Drug Administration, 2018, 2019, 2020). For example, the outbreak strains in three, separate *E. coli* O157:H7 outbreaks linked to romaine lettuce grown in Yuma, Arizona (Food and Drug Administration, 2018), and Salinas, California (Food and Drug Administration, 2019, 2020) were also isolated from canals or reservoirs used to source water for irrigation. Since outbreaks frequently result in consumer avoidance of the implicated food, there are both substantial public health and economic costs associated with outbreaks (Ribera et al., 2012; Hussain and Dawson, 2013; Hoffman, 2014). Heightened industry concerns surrounding the food safety risks associated with preharvest water use for produce production (Lewis Ivey et al., 2012; Schattman et al., 2018; Wall et al., 2019) are highlighted by a 2017 survey where 77% of the 155 Northeastern growers surveyed cited the need for irrigation practices that ensure produce safety as a key concern (Schattman et al., 2018). Since 70–80% of US growers rely on agricultural water (as opposed to rain) for irrigation (Rangarajan et al., 2002; Astill et al., 2018), water is integral to produce production. As such, science-based strategies for identifying produce safety hazards in agricultural water sources are needed.

To manage food safety hazards in agricultural water used for produce production, voluntary grower agreements, such as the Leafy Greens Marketing Agreement (Arizona Leafy Greans Marketing Agreement, 2012; California Leafy Greens Marketing Agreement, 2017) and federal legislation [i.e., Food Safety Modernization Act (FSMA); Food and Drug Administration, 2015] established microbial standards for surface water used in preharvest applications. For example, FSMA’s Produce Safety Rule states that growers must create a microbial water quality profile (MWQP) for each water source by collecting 20 samples over a 2–4 years period (Food and Drug Administration, 2015). The MWQP’s 90th percentile and geometric mean *E. coli* level must be <410 and <126 CFU/100-mL, respectively (Food and Drug Administration, 2015). However, meeting these standards has been repeatedly cited as a critical concern among industry stakeholders (Calvin et al., 2017; Astill et al., 2018; Wall et al., 2019). Following a summit focused on grower concerns about the FSMA standard, summit organizers summarized these concerns as centering on the (i) cost of meeting the standard, (ii) value of *E. coli-*based tests for assessing risk, (iii) lack of data supporting the standard’s water sampling frequency (e.g., 5 times/year for 4 years), and (iv) difficulties in accurately assessing risk due to the complexity of farm and freshwater environments (Wall et al., 2019). Since this summit, multiple studies have validated each of these concerns (e.g., Calvin et al., 2017; Havelaar et al., 2017; Truitt et al., 2018; Weller et al., 2020a). For example, several economic studies, including a study conducted by the USDA (Calvin et al., 2017), found that the costs used by the FDA to predict industry expenditures for complying with the FSMA standard were underestimated, and that water testing may be one of the largest FSMA-associated costs for growers (Astill et al., 2018). Other studies have highlighted the spatiotemporal variability in microbial water quality within and between sources, complicating interpretation of *E. coli*-based test results and suggesting that a one-size-fits-all standard fails to account for the complexity of freshwater ecosystems (Hipsey et al., 2008; Payment and Locas, 2011; Weller et al., 2020a). Recent studies have also shown that the testing frequency set by FSMA (20 samples over a 2–4 year period) means that a waterway meeting or exceeding the standard is largely dependent on when samples are collected and is not related to the presence of food safety hazards at the time of water use (Havelaar et al., 2017; Weller et al., 2020a). Conflicting data on the relationship between *E. coli* levels and foodborne pathogen presence has also cast doubt on the utility of *E. coli*-based water quality tests for identifying hazards in agricultural waterways (Edberg et al., 2000; Harwood et al., 2005; Wilkes et al., 2009; Bihn, 2011; Payment and Locas, 2011; Benjamin et al., 2013; McEgan et al., 2013a; Pachepsky et al., 2015; Antaki et al., 2016; Weller et al., 2020a). Indeed, the direction and strength of the relationship between *E. coli* levels and pathogen presence appear to be region, pathogen, and/or waterway-specific (Francy et al., 2013; McEgan et al., 2013a; Pachepsky et al., 2015; Bradshaw et al., 2016; Weller et al., 2020a). For instance, a Florida study found that the correlation between *E. coli* and *Salmonella* levels varied substantially between the 18 ponds sampled (correlation coefficients ranged between 0.0 and 0.7; McEgan et al., 2013a). Overall, the literature suggests that alternatives to *E. coli* based-water quality testing and standards are needed for assessing food safety hazards in agricultural water used for produce production.

Past studies have suggested that physicochemical water quality parameters (e.g., turbidity) could be used as supplementary indicators (i.e., along with *E. coli* levels) of food safety hazards being present in agricultural water (Harwood et al., 2005; Bihn, 2011; Pachepsky et al., 2015). However, a common refrain within the food safety community is that we cannot “test our way to food safety,” and that alternatives to test-based management strategies are needed. A previous study that sampled six Florida ponds used to source water for produce production, used support vector machines (SVM), k-nearest neighbor (kNN) and neural net learners to develop univariable models to predict *Salmonella* presence or absence (i.e., nine models per algorithm; Polat et al., 2019). This study demonstrated the potential utility of predictive models for managing enteric pathogen contamination of agricultural water in the Southeast (Polat et al., 2019). Similarly, studies conducted in the Northeastern United States have developed and validated classification tree-based models for predicting *Listeria monocytogenes*, and *Listeria* spp. presence in produce field soils (Strawn et al., 2013; Weller et al., 2016). The findings from Polat et al. (2019) and the New York studies (Strawn et al., 2013; Weller et al., 2016) suggest that similar approaches could be used to predict when and where enteric pathogens are likely to be present in Northeastern streams used to source water for produce production. The primary aim of this study was to determine if machine learning could be used to develop models that accurately predict enteric pathogen presence in agricultural water sources in a different produce-growing region (the Northeast). Due to the costs associated with collecting certain data types (e.g., microbial water testing; Calvin et al., 2017), a secondary aim of this study was to (i) assess the relative information gain associated with using different data types to build predictive models, and (ii) determine if accurate models could be built without using microbial data. This study also aimed to underscore the limitations and strengths of various machine-learners and provide guidance on how these learners can be used in future applied environmental microbiology studies. It is important to emphasize that this study was conducted not to develop models that could be deployed in the field. Instead, it is a proof-of-concept that can be built upon to develop field-ready models once larger, multi-region datasets become available.

## MATERIALS AND METHODS

### Study Design

**FIGURE 1 |:**
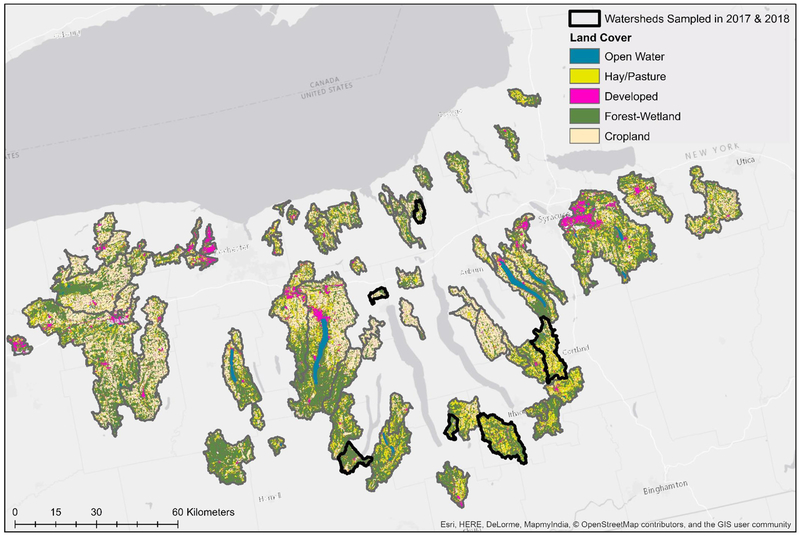
Map of land cover in watersheds sampled in both study years (No. = 6), and watersheds sampled only in 2018 (No. = 62).

Two previously published datasets collected in 2017 (Weller et al., 2020a) and 2018 (Weller et al., 2020b), respectively, were used in the analyses reported here (Table 1). While the data presented here were reported previously (Weller et al., 2020a,b), those manuscripts focused on (i) assessing the impact of sampling methods on pathogen detection, and (ii) characterizing associations between microbial water quality, and other environmental features (e.g., rainfall, turbidity). In contrast, the objectives of this study, including the development and comparison of predictive models using multiple machine learners, is novel to the study reported here. The only differences in sampling and laboratory protocols between the two datasets are (i) the number of streams sampled, and (ii) the frequency with which sampling occurred. Specifically, in 2017 six streams were sampled between 15 and 34 times each (*N* = 181 samples total; Weller et al., 2020a), and in 2018 sixty-eight streams were sampled between 2 and 3 times each (*N* = 191 samples total; Weller et al., 2020b; Table 1; Figure 1). At each sampling, separate 10-L grab samples (GS) were collected and tested for the *eaeA* and *stx* genes (biomarkers for pathogenic *E. coli*; Smith et al., 2009), and *Salmonella*. A one 1-L GS (for *E. coli* enumeration) was also collected. At each sampling, physicochemical water quality data were also collected as previously described (Weller et al., 2020a,b). Gloves (Nasco, Fort Atkinson, WI) were changed for each sample collected, and sampling materials were sprayed with 70% ethanol between all sample collections. All samples were transported on ice, stored at 4°C, and processed <18 h of collection. In lab, each 10-L GS was filtered using a modified MS (mMS) as previously described (Sbodio et al., 2013; Weller et al., 2020a,b). After filtration, each mMS was transferred to a sterile Whirl-Pak and processed as described below. A 100-mL aliquot of the 1-L GS was used for *E. coli* enumeration, which was performed using the Colilert Quanti-Tray 2000 kit (IDEXX, Westbrook, ME) per manufacturer instructions and as previously described (Weller et al., 2020a,b).

### Metadata Acquisition

**FIGURE 2 |:**
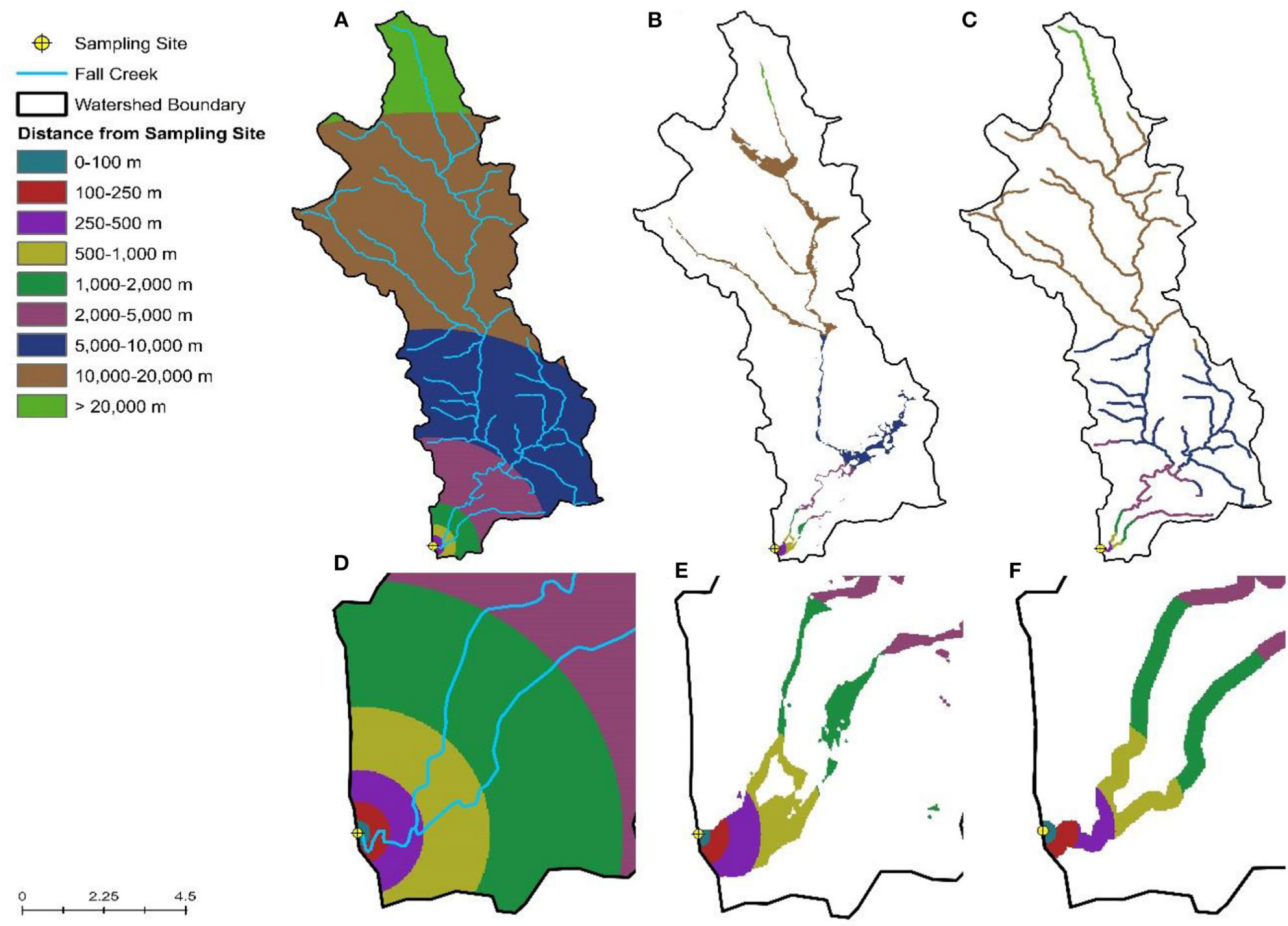
Visualization of the inverse distance weighting approach used to calculate the percent of the watershed **(A)**, floodplain **(B)**, and riparian buffer **(C)** under different land uses. **(D–F)** Provide a close-up view of **(A–C)**, respectively, for areas near the sampling site.

Spatial data were obtained from publicly-available sources as previously described (Weller et al., 2020b; Supplementary Table 1). Watershed delineation and all other spatial analyses were performed using ArcGIS version 10.2 and R version 3.5.3. Inverse-distance weighting (IDW) was used to characterize land cover within watersheds, while accounting for the fact that areas closer to the sampling site are more likely to impact water quality than areas further upstream (King et al., 2005). By weighting land use based on distance to the sample site, this also reduces the noise in the land-use data that would be present due to differences in watershed size. Briefly, land cover percentages for each of the following distance intervals around the sampling site were calculated: 0–100, 100–250, 250–500, 500–1,000, 1,000–2,000, 2,000–5,000, 5,000–10,000, 10,000–20,000, and >20,000 m; intervals were constrained by watershed boundaries (Figure 2). We then adapted the equation from King et al. (2005) to calculate the inverse-distance weighted percentage for each land cover class. In addition to characterizing land cover within the sampled watersheds, we also calculated the IDW percentage of each land cover in the flood plain and within the stream corridor. We also determined if specific features were present upstream of the sampling site as well as the density of these features as previously described (Supplementary Table 1; Weller et al., 2020b).

Weather data were obtained from the closest NEWA station (newa.cornell.edu) to each sample site. The nearest station was determined by drawing Thiessen polygons around each station. The average distance of the stations to the sites was 8.9 km (range = 0.4–25.5 km). If a sensor malfunctioned then data for that parameter from the next nearest station was used while the malfunction persisted. Average air temperature, average solar radiation, and total rainfall were calculated using nonoverlapping time periods (0–1, 1–2, 2–3, 3–4, 4–5, 5–10, 10–20, and 20–30 d before sample collection).

### Salmonella and *eaeA*-*stx* Detection

*Salmonella* enrichment and isolation were performed as previously described (protocol in Supplemental Materials of Weller et al., 2020b, and at github.com/wellerd2/Laboratory-Protocols). Briefly, 225 mL of buffered peptone water supplemented with 5 mg of novobiocin was added to each Whirl-pak. Following incubation at 35°C for 24 h, *Salmonella* negative samples and presumptive *Salmonella* positive samples were identified using real-time BAX *Salmonella* assays (Hygiena, Wilmington, DE). BAX negative samples were considered *Salmonella* negative, while BAX positive samples were culture-confirmed as *Salmonella*-positive as previously described (Weller et al., 2020b).

The *eaeA* and *stx* genes are considered biomarkers for enteropathogenic *E. coli*, Shiga-toxin producing *E. coli* (STEC), and/or enterohemorrhagic *E. coli*. A PCR-screen for these genes was performed using a real-time BAX STEC) assay (Hygiena) per manufacturer’s instructions. Samples that were positive by PCR-screen for both genes were considered positive for pathogenic *E. coli* in the study reported here.

### Statistical Analyses

All analyses were performed in R (version 3.5.3; R Core Team, Vienna, Austria). The learners used here were selected to include common learners (e.g., regression, tree-based), novel learners that appear promising (e.g., Node Harvest), and learners used in past produce safety research (e.g., classification trees; Meinshausen, 2010; Deng and Runger, 2013; Strawn et al., 2013). Model training and testing were performed using the mlr package^1^. The 2018 dataset (Weller et al., 2020b) was used for model training and the 2017 dataset (Weller et al., 2020a) was used for model testing (Table 1). Separate models were developed to predict the presence or absence of *Salmonella*, and of the *eaeA-stx* genes. Repeated 3-fold cross-validation was used to tune hyperparameters and perform cross-validation. Tuning was performed to optimize the area under the curve (AUC). Following tuning, models were trained and predictive performance assessed using the test data. Since the values of several performance measures (e.g., sensitivity) are dependent on the probability threshold, the threshold was also tuned to optimize the kappa score. During model development, four types of features were considered: microbial water quality, physicochemical water quality, spatial, and weather (Supplementary Table 1). Models built using all four feature types were designated “full models,” while models built using one, two, or three of these feature types were designated “nested models.” Prior to model development, the training and test data were merged, and all features were centered and scaled. The training and test data were then split back into separate datasets. It is better practice to first standardize the training data, and then use the means and standard deviations from the training data to standardize the testing data; future studies, particularly studies aimed at developing model-based tools for use on farms, should consider using this approach when standardizing training and testing data.

The 20 learners used to develop the full models can be grouped into tree-based learners, forests, instance-based learners, Bayesian learners, regressions, rule-based learners, and support vector machines (SVM). Although 20 learners were used to build the full models, four variants of SVMs were implemented, resulting in 23 full models per outcome. It is important to note that several of the learners used here are quite similar, and would be redundant in a study that aimed to develop a field-ready predictive model. However, since one of the aims of this paper is to underscore the limitations and strengths of various machine-learners and provide guidance on how these learners can be used in future applied environmental microbiology studies, a large number of learners were used. Separately from the full models, the nested models were developed to assess the relative information gain associated with using different types of features for model training. Prior to the start of the study, six learners were randomly selected from the 23 learners used for full model development. Nine nested models were then built for each of these six learners using: microbial features; microbial features and turbidity; physicochemical water quality features; weather features; microbial and physicochemical water quality features; microbial and weather features; microbial, physicochemical water quality, and weather features; physicochemical water quality and weather features; and spatial features. Performance measures for each model were calculated and visualized graphically. The top-ranked models for each outcome were identified by (i) ranking models based on positive and negative likelihood ratio, diagnostic odds ratio, AUC, F1-score, and kappa score, and (ii) averaging each model’s rank for these six measures. A higher rank indicated better performance; models that tied were assigned the same rank. The performance of the top-ranked *Salmonella* and *eaeA-*stx models were visualized using density, ROC, and split quantiles plots. Explanations on how to interpret these plots are included in the figure legends.

### Baseline Models

Baseline models were created using current or proposed agricultural water quality standards (Steele and Odumeru, 2004; Food and Drug Administration, 2015; Uyttendaele et al., 2015). Each standard is based on an acceptable level of an indicator organism being present in the sample. Samples above this cut-off are non-compliant and samples below the cut-off are compliant. The cut-offs considered were: 126, 235, 410, and 1,000 MPN of *E. coli/*100-mL, and 1,000 MPN of coliforms/100-mL (Steele and Odumeru, 2004; Food and Drug Administration, 2015; Uyttendaele et al., 2015). Samples with *E. coli* levels below the cut-off were predicted to be negative for the target pathogen (*Salmonella* or *eaeA*-*stx*), while samples above the cut-off were predicted to be positive. The epiR and exact 2 × 2 packages were used to calculate performance measures for each baseline model. Boxplots were used to visually compare *E. coli* levels between pathogen positive and negative samples, and between the training and test data.

### Tree-Based

Three tree-based learners were used: classification trees (CART), conditional inference trees (cTree), and evolutionary optimal trees (evTree). Due to their interpretability and ability to handle non-linear relationships and interactions, tree-based models are often used to characterize associations in datasets that may not meet parametric assumptions (e.g., Strawn et al., 2013; Bradshaw et al., 2016; Weller et al., 2016). Briefly, tree-based learners hierarchically partition data into homogenous subsets; for our data this meant generating terminal nodes that consisted of either pathogen positive or negative samples. CART and cTree generate locally-optimal trees via forward stepwise processes, while evTrees generate globally optimal trees (see Grubinger et al., 2014 for differences between locally and globally optimal trees). The CART, cTree, and evTree models were implemented here using the rpart, party, and evtree packages, respectively. Maxdepth (maximum depth to which a tree can be grown) and minbucket (minimum number of observations allowed in terminal nodes) were tuned for all 3 learners. To minimize the potential for overfitting, complexity parameters were tuned during CART (cp parameter) and evTree (alpha parameter) implementation, and mincriterion was set to 0.95 during cTree implementation.

### Forests

One of the disadvantages of tree-based learners, is that small changes in the training data (e.g., due to sampling-induced variability) can produce large changes in model fit (Breiman, 1996). Ensembles of tree-based learners, or forests, were developed to overcome this limitation by aggregating predictions across thousands of trees. Three random forest learners were used here: the original algorithm proposed by Breiman (2001, RF), regularized random forest (regRF; Deng and Runger, 2012, 2013), and conditional random forest (condRF; Strobl et al., 2009). RF models are ensembles of CART trees, and cannot handle correlated features (Strobl et al., 2007, 2008, 2009). condRF was developed to overcome these limitations, and is an ensemble of cTrees (Strobl et al., 2007, 2008, 2009). While regRF was not developed to expressly deal with the limitations of RF models, regRF does incorporate a feature selection step, which reduces (i) the impact of correlation on variable importance estimates, and (ii) redundancy in the overall feature set (Deng and Runger, 2012, 2013). The minbucket and mtry parameters (number of features included in each random subset used for building splitting rules) were tuned for all 3 random forest learners. The coefficient of regularization was tuned when implementing regRF, and mincriterion was tuned when implementing condRF.

Boosting is another way of generating forests. In a boosted forest, the input data used to train each new tree are the residuals from the antecedent tree. New trees are added sequentially until the addition of a new tree fails to improve performance. Here we used extreme gradient boosting (xgBoost; Chen and Guestrin, 2016), and tuned hyperparameters that control: (i) learning rate and overfitting; (ii) if new splits are added to each tree; (iii) the number of rounds of boosting performed; (iv) maxdepth; (v) the proportion of data used to build each tree; (vi) the number of features considered when building each tree; and (vii) regularization. xgBoost was the most computationally intensive learner used here, and took multiple days to complete.

The main trade-off between tree-based learners and forests is between interpretability and performance; tree-based models are more interpretable but less accurate than forests (Table 2). Node harvest was developed to reconcile these trade-offs (Meinshausen, 2010). When creating a node harvest model, a large set of trees is generated using a RF algorithm. However, unlike a true RF, only a subset of the training data is used to grow each tree. Nodes from each tree are then sequentially extracted, and added to a “node set” (Meinshausen, 2010; Van De Put, 2017). A subset of nodes is then selected for inclusion in the final learner and assigned weights. Model predictions are based on the weighted average of the proportion of pathogen-positive training samples in each node whose rules a novel observation meets (for examples see Van De Put, 2017). For the node harvest models reported here we tuned: (i) the minimum number of training data samples to use when building each tree and (ii) maxdepth.

### Instance-Based Learners

Three instance-based learners were used here: k-nearest neighbor (kKNN), weighted k-nearest neighbor (wKNN), and random k-nearest neighbor (rKNN) using the kknn, rknn, and mlr packages. Instance-based learners use the *k* training samples whose characteristics are most similar to a new sample to predict the pathogen status of this new sample. As a result, the accuracy of instance-based learners are highly dependent on the value of *k*. The predicted pathogen status for the novel sample is determined using either majority-voting or a probabilistic approach (e.g., kernel density estimation; Hechenbichler and Schliep, 2004). A disadvantage of majority-voting, which is used by the rKNN algorithm, is that probability-based measures like AUC cannot be calculated. A disadvantage of both kKNN and rKNN is that all *k* neighbors contribute equally to a prediction even though these *k* neighbors may vary in how similar they are to the novel sample. To overcome this disadvantage, the wKNN algorithm converts the distance between a new sample and each neighbor to a similarity measure, which is used to weight the contribution of that neighbor to the prediction. Since there are multiple ways to calculate wKNN weights (see Hechenbichler and Schliep, 2004), the weighting algorithm was tuned in current study. For all three instance-based learners implemented here, the value of *k* was tuned. When implementing the kKNN and wKNN learners, the distance metric calculated (Euclidean or Manhattan) was also tuned.

rKNN is an ensemble learner that consists of *r* kKNN models, each built using a random subset of features (Li, 2009), which should make rKNN models more robust to noise in the dataset than kKNN models. The number of kKNNs used in the rKNN models implemented here was set to 20,001. Since rKNN categorizes samples as positive or negative for the target (instead of predicting the probability of the sample being positive), (i) AUC could not be calculated so the kappa score was optimized during rKNN hyperparameter tuning, and (ii) the probability threshold was not tuned when calculating rKNN performance measures.

There are several advantages to instance-based learners, including the fact that they (i) are non-parametric and do not make assumptions about the distribution of features or residuals, and (ii) use the raw training data (as opposed to a discriminative function) to make predictions. The latter allows the model to be updated as new data become available (Li et al., 2011). However, instance-based learner performance is affected by (i) biases and noise in the training data (e.g., due to measurement error), and (ii) the features used (e.g., use of irrelevant features increases misclassification rate). As a result, performing feature selection prior to implementing instance-based learners is sometimes recommended (Li et al., 2011). Since kKNN was one of the learners used to build the nested models, additional feature selection was not performed in the present study but should be considered in future studies. It is important to note that feature selection is either not required or performed automatically for many of the other learners considered in the current study (Table 2).

### Naïve and Semi-Naïve Bayesian Learners

Two Bayesian learners were implemented here: naïve Bayes and random Ferns (rFerns). Naïve Bayes are simple models based on the assumption that each feature contributes independently to the probability of a novel sample being pathogen positive (Kuhn and Johnson, 2016). In its simplest form (where there is one feature) Naïve Bayes works by predicting the probability of a novel sample being pathogen positive given that a different event occurred first (e.g., the sample was collected from a stream with a sandy bottom; Kuhn and Johnson, 2016). The only parameter tuned when implementing Naïve Bayes controlled Laplace smoothing and regularization.

rFerns is a non-hierarchical ensemble of Bayesian learners; the constituent learners in rFerns models are called ferns as opposed to trees (Özuysal et al., 2010; Kursa, 2014). Each fern consists of a series of binary rules built using an arbitrary set of features (Özuysal et al., 2010; Kursa, 2014). The pattern of Yes/No responses for each fern is used to generate a distribution (Özuysal et al., 2010; Kursa, 2014). This distribution is then used to estimate the probability of a new sample being pathogen positive given the pattern of Yes/No responses for each single fern (Kursa, 2014). To prevent overfitting, rFerns randomly generates the thresholds used to dichotomize continuous and multi-class categorical features (Kursa, 2014). The only parameter tuned when creating the rFerns models controlled the maximum number of rules included in each fern. The number of ferns generated per model was set to 20,001. Like rKNN, rFerns categorizes samples as pathogen positive or negative, and (i) AUC could not be calculated and kappa score was optimized during hyperparameter tuning, and (ii) the probability threshold was not tuned when calculating performance measures.

### Penalized Regression

Three penalized regression learners were used here: lasso, ridge, and elastic net. Regression analysis is commonly used to characterize the association between environmental factors and foodborne pathogen detection (e.g., Benjamin et al., 2015; Ceuppens et al., 2015; Weller et al., 2015, 2020b). A key advantage of regression over “black-box” methods (e.g., RF, SVM) is that the output from regression is highly interpretable (Kuhn and Johnson, 2016). However, use of correlated features when implementing traditional regression approaches results in overestimation of variance (Kuhn and Johnson, 2016). Penalized regression applies a penalty to control the magnitude of the parameter estimates and account for correlation between features. In ridge regression a penalty is added to the sum of the squared regression parameters so that estimates become smaller as the penalty becomes larger. As a result, ridge regression does not perform feature selection, instead ridge regression shrinks the coefficient estimates of features that are not associated with the outcome close to 0. In contrast, lasso and elastic net regression incorporate feature selection. Lasso regression uses a similar penalty to ridge regression but allows coefficient estimates to be 0. Essentially ridge regression shrinks the parameters of correlated features toward each other allowing each parameter to borrow strength from the other, while lasso regression only retains one feature from a group of correlated features (Friedman et al., 2010). Elastic net combines the strengths of lasso and ridge regression by using a ridge-type penalty for regularization and a lasso-type penalty for feature selection. In the present study, the cv.glmnet function (glmnet package) was used to implement all three regression learners. For all three regression learners, the number of cross-validated folds performed internally was set to 10, and the “s” parameter (which determines if the model with the min. mean cross-validated error or a model within one standard error of the min. is retained as the final model) was tuned. The lambda parameter was also tuned for all three models with the package default of 100 potential lambda values being considered. For ridge and lasso regression the alpha parameter was set to 0 and 1, respectively, while the alpha parameter was tuned for elastic net regression.

### Rule-Based Learners

Three rule-based learners were implemented here using the RWeka package: one propositional rules (OneR), partial decision lists (PART), and repeated incremental pruning to produce error reduction (JRip). While tree and rule-based learners are similar conceptually, tree-based learners take a divide-and-conquer approach and rule-based learners take a separate-and-conquer approach. Divide-and-conquer learners create hierarchical rules that make terminal nodes as homogenous as possible using all input data. Separate-and-conquer learners recursively create individual rules, remove observations in the training data that were correctly classified by this rule, and then create a new rule to classify the remaining observations. OneR uses one feature to generate a single rule (basically a decision tree with a single split; Holte, 1993; Parsania et al., 2014). OneR was developed as a baseline learner; for more complex, less-interpretable learners to be useful that learner should perform better than the OneR model (Holte, 1993; von Jouanne-Diedrich, 2017). In the current study minbucket was the only parameter tuned when implementing OneR.

The JRip learner subsets the training data into growing and pruning data. A series of initial rules are then created using the growing data. These rules are repeatedly simplified to yield the greatest reduction in error for predictions made on the pruning data (Cohen, 1995). The post-pruning rule set is then optimized by adding new rules, or by replacing or revising existing rules. Growth, pruning, and optimization are then recursively repeated (Cohen, 1995; Frank and Witten, 1998). When implementing JRip the number of folds used to split the training data into growing and pruning subsets, the minimum number of observations allowed in children nodes, and the number of optimization runs to perform were tuned.

PART was developed to overcome several disadvantages of JRip (e.g., tendency for overpruning; Frank and Witten, 1998). As a result, JRip is a true rule-based learner that uses a separate-and–conquer approach (Cohen, 1995), while PART combines the divide-and-conquer and separate-and-conquer approaches. Briefly, when implementing PART, a partial decision tree is built using all observations in the training set. The leaf within the tree that correctly classified the most observations is then converted into a rule, and the rest of the tree is discarded (Frank and Witten, 1998). This process is then recursively repeated using only those training data points that were not classified correctly by antecedent rules. When implementing PART the threshold used for pruning the trees and the minimum number of observations allowed in a leaf were tuned. The number of separate growing and pruning sets was set to 3.

### Support Vector Machines (SVM)

SVMs were implemented here using the e1071 package. SVMs work by transforming the training data, and then finding the optimal hyperplane in *N*-dimensional space that maximally separates the training data into pathogen negative and positive samples. By transforming the data, SVMs can be extended to patterns that are not linearly separable; four kernels were considered here when transforming the data: linear, radial, sigmoid, and polynomial (i.e., 4 SVM models per pathogen). The radial, sigmoid and polynomial kernels use different transformations to map the data to higher (polynomial) or lower (radial) dimensional space. The number of parameters tuned during SVM implementation reflects the dimensionality of the kernel. Regardless of dimensionality, a penalty parameter that controls the trade-off between the smoothness of the hyperplane’s decision boundary and classification accuracy was tuned. How close a sample needs to be to the hyperplane to influence it was tuned when implementing a SVM with a non-linear kernel, while a parameter that allows the hyperplane to by non-symmetric was tuned for SVMs with sigmoid and polynomial only. The degree of the polynomial function was tuned for the polynomial SVMs only.

## RESULTS AND DISCUSSION

In total, 82 models per target (*Salmonella* and *eaeA-stx*) were developed here, including 23 full models (models built using all four feature types; Supplementary Table 1), 54 nested models (models built using between one and three feature types), and five baseline models. Previously published datasets collected in 2017 (Weller et al., 2020a) and 2018 (Weller et al., 2020b) were used to test and train the models, respectively (Table 1). The prevalence of *Salmonella* and the *eaeA-stx* genes was approx. the same in 2017 and 2018 (Table 1). While the distribution of *E. coli* levels was also approx. the same in 2017 and 2018 (Table 1; Figure 2), there were several outlier values in the 2018 data. These outliers may be a product of sampling differences between the two datasets. Specifically, six streams were intensely sampled between May and August 2017 while 68 streams were sampled between April and October 2018. By using two independently collected datasets, models could be both trained and validated here, facilitating assessment of model performance. By comparison, past studies that developed models to predict foodborne pathogen presence in preharvest environments either did not perform model validation (e.g., Bradshaw et al., 2016), or published validation results separately from the paper describing the predictive model (e.g., Strawn et al., 2013; Weller et al., 2016). However, the small number of streams represented in the test data (*N* = 6) means that test results reported here may be limited in generalizability. Such concerns are exacerbated by the size of the training and test datasets, and the fact that the datasets represent a single produce-growing region (Upstate New York; Figure 1), one agricultural water type (streams but not canals or ponds), and a single growing season (2017 or 2018). However, these limitations are mediated by the large number of streams represented in the training data (*N* = 68). In fact, the number of streams sampled in 2018 is substantially greater than past studies that developed models to predict foodborne pathogen presence in farm and freshwater environments (Efstratiou et al., 2009; Shiels and Guebert, 2010; Francy et al., 2013; McEgan et al., 2013a; Strawn et al., 2013; Bradshaw et al., 2016; Weller et al., 2016; Polat et al., 2019). Additionally, these past studies often used less robust modeling approaches (e.g., unpenalized regression, CART) and/or a limited set of features (e.g., univariable models built using a single microbial or physicochemical water quality feature) than the study reported here (Efstratiou et al., 2009; Shiels and Guebert, 2010; Francy et al., 2013; McEgan et al., 2013a; Strawn et al., 2013; Bradshaw et al., 2016; Weller et al., 2016; Polat et al., 2019). It is also important to reiterate here that the aim of this study was not to develop predictive models that could be used by growers to guide on-farm decision-making, instead this was a proof-of-concept study that can be built upon once larger, multiyear, and multi-region datasets become available. Specifically, we aimed to generate findings to provide guidance on the learners (e.g., support vector machines, ensemble learners) and data types (i.e., microbial for *eaeA-stx*, and spatial for *Salmonella*) that should be the foci of future efforts. For this reason, and because many of the methods used here are black-box approaches, neither feature importance nor associations between features, and *eaeA-stx* and *Salmonella* detection are reported here; instead, these results can be found in papers previously published using these datasets (Weller et al., 2020a,b).

### Water Quality Standards Based on Binary *E. coli* Cut-Offs Alone May Not Be Suitable for Assessing Food Safety Hazards in Agricultural Water

**FIGURE 3 |:**
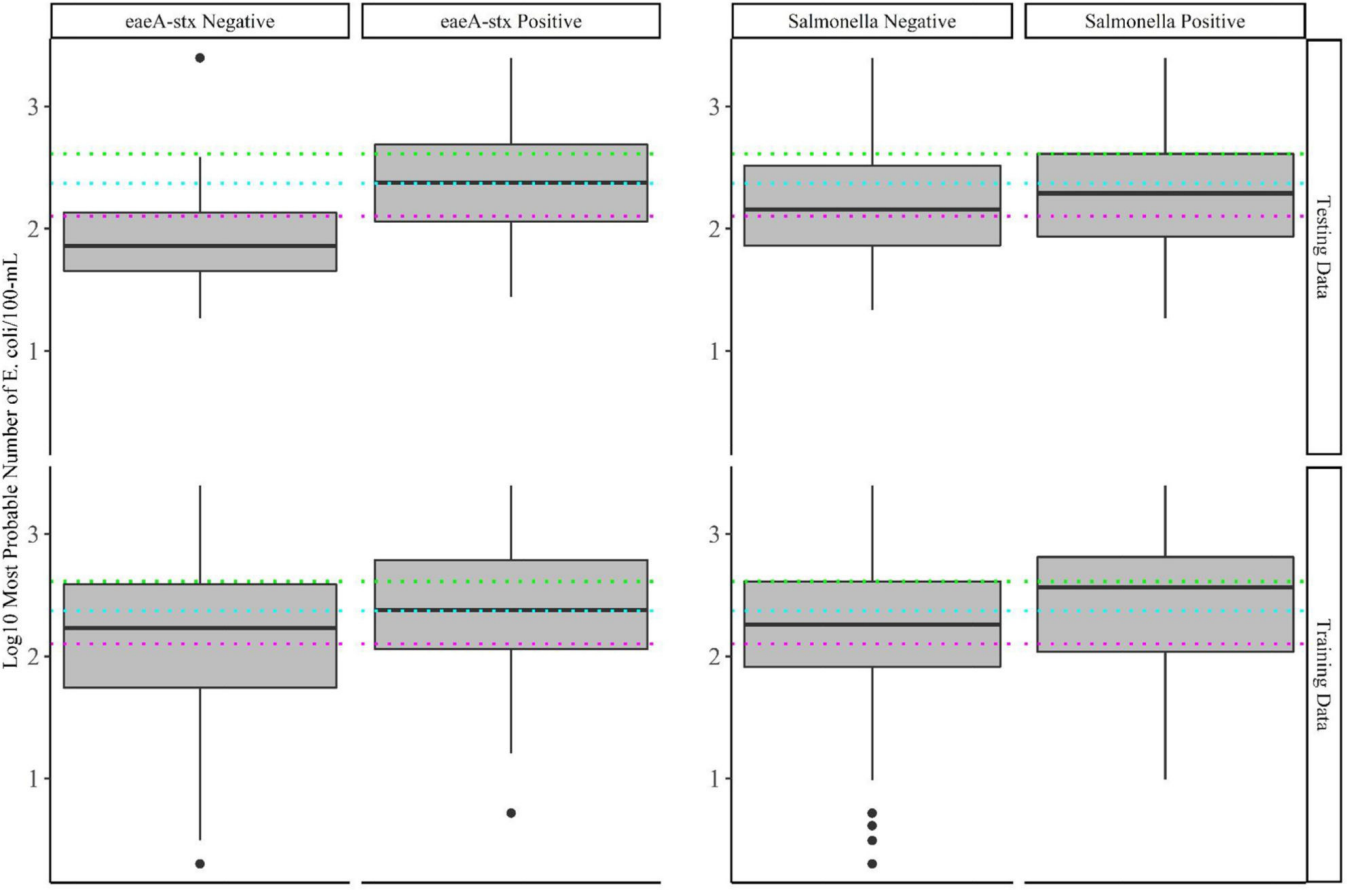
Log 10 *E. coli* levels in training and test data samples that tested positive and negative for *eaeA-stx* and *Salmonella*. The colored lines represent the thresholds for agricultural water that were considered during development of the US Food Safety Modernization Act’s Produce Safety [126 MPN/100-MmL (pink), 235 MPN/100-mL (blue), and 410 MPN/100-mL (green)].

Baseline models were created using five current or proposed microbial water quality standards (Steele and Odumeru, 2004; Food and Drug Administration, 2015; Uyttendaele et al., 2015), which are based on an acceptable level of *E. coli* or coliforms being present in a sample. The five cut-offs considered were: 126, 235, 410, and 1,000 MPN of *E. coli/*100-mL, and 1,000 MPN of coliforms/100-mL. While models based on 1,000 MPN of *E. coli* and 1,000 MPN of total coliforms per 100-mL were not able to accurately differentiate *eaeA-stx* positive and negative samples, the three remaining cutoffs (126, 235, or 410 CFU/100-mL of *E. coli*) were able to accurately differentiate *eaeA-stx* positive and negative samples in the test dataset (Figure 3; Supplementary Table 2). In fact, the 126 MPN model was among the five top-ranked *eaeA-stx* models (Supplementary Table 2; Supplementary Figure 3). Conversely, all five baseline models were unable to accurately differentiate *Salmonella* positive and negative samples in the test dataset (Figure 3; Supplementary Table 2). Findings based on the test dataset are consistent with some studies that found evidence of a relationship between generic *E. coli* levels and pathogenic *E. coli* presence, and/or failed to find evidence of a relationship between generic *E. coli* levels and *Salmonella* presence (Benjamin et al., 2013; Antaki et al., 2016; Weller et al., 2020a). However, when predictions were made on the training data (data used for model building) the baseline models were unable to accurately differentiate *eaeA-stx* positive and negative samples. For instance, the 126-MPN model, which was the most accurate baseline *eaeA-stx* model when predictions were made on the test data [False Positive Rate (FPR) = 0.27; Kappa = 0.44; Supplementary Table 2] was unable to correctly identify *eaeA-stx* negative samples in the training data (FPR = 0.55; Kappa = 0.16). Similar results were found for the 410-MPN *eaeA-stx* model (Training FPR = 0.24; Test FPR = 0.02), and the 235-MPN *eaeA-stx* model (Training FPR = 0.39; Test FPR = 0.16; Figure 3). This discrepancy in baseline model performance is not unexpected given the conflicting data on the relationship between *E. coli* levels and pathogen presence in the literature (Edberg et al., 2000; Harwood et al., 2005; Smith et al., 2009; Wilkes et al., 2009; Bihn, 2011; Payment and Locas, 2011; Benjamin et al., 2013; Economou et al., 2013; McEgan et al., 2013a; Pachepsky et al., 2015; Antaki et al., 2016; Weller et al., 2020a) Indeed, multiple studies that examined the relationship between *E. coli* levels and pathogen presence in agricultural water sources suggest that the direction and strength of this relationship may be region, weather, water source, and/or pathogen-specific (Francy et al., 2013; McEgan et al., 2013a; Pachepsky et al., 2015; Bradshaw et al., 2016; Weller et al., 2020a). It is also important to note, that these observations are consistent with the fact that *E. coli* is (i) an indicator of fecal contamination and not an index organism for a specific pathogen, and (ii) multiple studies have shown that generic *E. coli*, pathogenic *E. coli*, and *Salmonella* can naturalize in non-host environments, including water (Hendricks, 1967; Byappanahalli et al., 2003; Whitman et al., 2003; Busta et al., 2006; Ksoll et al., 2007; Nautiyal et al., 2010; Goto and Yan, 2011; McEgan et al., 2013b; NandaKafle et al., 2018). Viewed in this context, and given the small number of streams represented in the test data, our findings make sense. With a small number of streams, the test dataset is more likely to be biased by features unique to a single stream, which is why it was used for model testing and not training in this study. Indeed, if bovine fecal inputs into a stream were regularly contaminated by fecal *eaeA-stx* and generic *E. coli*, the signal from this stream would be diluted by the other 67 training data streams but less so by the other 5 test data streams. Indeed, the presence of cattle operations immediately upstream of two of the test data streams, could explain the difference in baseline model performance when predictions were made on the training and test data. As such, our findings are illustrative of the impact of study design on data interpretation and generalizability, and of the importance of selecting representative training and test data sets when building and testing predictive models. Due to the limited number of multi-regional, multi-year studies that surveyed food safety hazards in agricultural water, additional surveys are needed before predictive models can be developed, validated, and deployed to support on-farm decision-making.

Since the baseline models were built using current or proposed microbial water quality standards (Food and Drug Administration, 2015; California Leafy Greens Marketing Agreement, 2017), our findings also support previous studies’ conclusions that agricultural water quality standards based on binary *E. coli* cut-offs alone may not be a reliable indicator of food safety hazards presence in agricultural water (Duris et al., 2009; Havelaar et al., 2017; Weller et al., 2020a). In drawing this conclusion, it has to be noted that the baseline models make predictions using a single sample (i.e., a snapshot in time), while most regulations apply the cut-offs to a profile consisting of multiple samples. For example, the FSMA standard use cut-offs of 126 and 410 MPN/100-mL but these cut-offs are applied to the geometric mean and statistical threshold value of 20 samples collected over 2–4 years, respectively. As a result, our findings are not directly comparable to existing water quality standards, but, when viewed in the context of the existing literature, do provide hypotheses about the utility of these standards that should be examined in future simulation studies. Specifically, this study raises the hypothesis that water quality standards based on binary *E. coli* cut-offs alone may not be appropriate for managing food safety hazards in agricultural water, and that alternative or supplementary management strategies (e.g., predictive models) are needed.

### Predictive Models May Be Useful for Identifying When and Where Food Safety Hazards Are Present in Agricultural Water Sources Used for Produce Production

**FIGURE 4 |:**
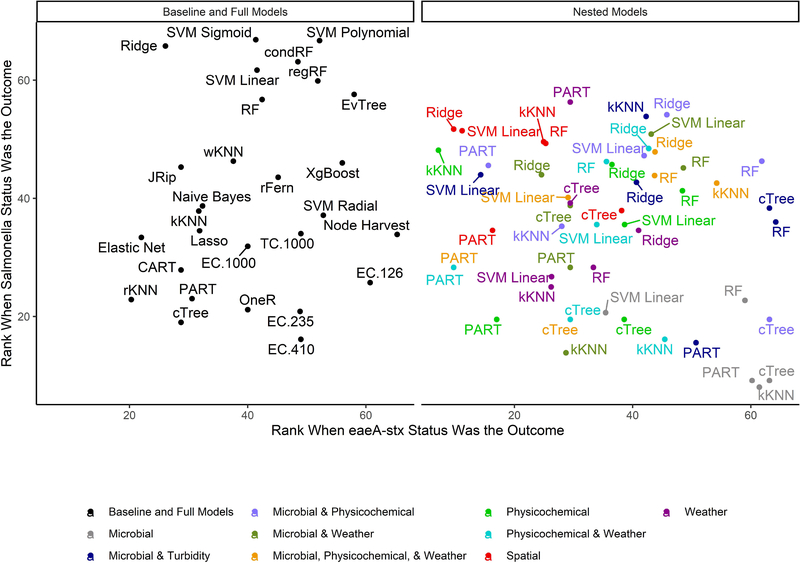
Mean rank (0 = worst; 65 = best) of each learner-data combination for each outcome. To facilitate readability, full and baseline models are depicted in a separate facet from the nested models, which were built using a subset of features. For baseline models, the letters refer to the organism the cutoff is based on (EC = *E. coli*, TC = total coliforms), and the number refers to the cut-off value (e.g., EC.126 is based on a cut-off of 126 MPN of *E. coli*/100-mL). Models that were able to accurately predict both *Salmonella* and *eaeA-stx* presence appear in the top right corner of each facet, while poor performing models appear in the bottom left of each facet.

**FIGURE 5 |:**
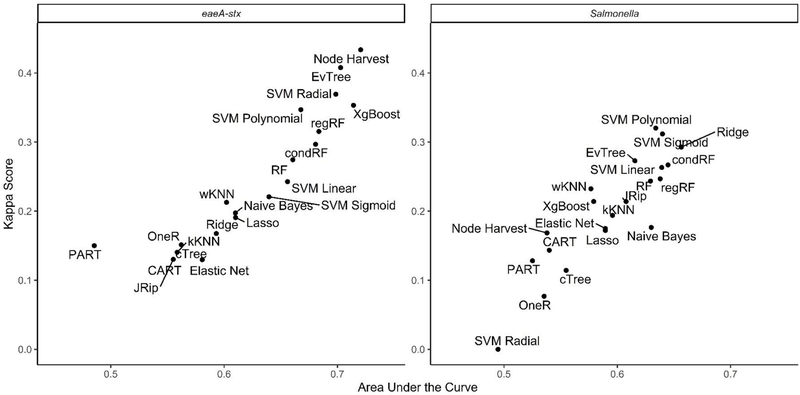
Plot showing kappa score and area under the curve for the full models.

When all data types were used in model development (i.e., the full models) the top-ranked model for *eaeA-stx* detection was built using the node Harvest algorithm [AUC = 0.72; Diagnostic Odds Ratio (DOR) = 3.8; Se = 0.89; Sp = 0.52; Figures 4, 5, 8, Supplementary Figures 1–3; Supplementary Table 3]. The top-ranked *Salmonella* full models were built using the SVM with a sigmoidal kernel (AUC = 0.64; DOR = 4.4; Se = 0.80; Sp = 0.48) and SVM with a polynomial kernel (AUC = 0.63; DOR = 5.7; Se = 0.86; Sp = 0.52; Figures 4, 5, 7, Supplementary Figures 1–3; Supplementary Table 2). Both the *Salmonella* SVMs and *eaeA-stx* node Harvest full models outperformed the baseline models built using current or proposed water quality standards and the OneR models (Figure 4; Supplementary Tables 2, 3). In fact, the 410-MPN baseline model was the lowest ranked *Salmonella* model while the 235-MPN baseline model was third to last. This finding suggests that predictive models, like those developed here, may be useful (i.e., as an alternative or supplementary strategy to microbial water quality testing) for identifying when and where food safety hazards are likely to be present in agricultural surface water. This conclusion is consistent with the findings from the only other study that developed models to predict foodborne pathogen presence in surface water sources used for produce production (Polat et al., 2019). This study used three learners, and nine water quality and weather features to predict *Salmonella* contamination in Florida irrigation ponds (Polat et al., 2019). The Florida study (Polat et al., 2019) found that, instance-based models could correctly classify up to 77% of training samples and 59% of test samples as *Salmonella* positive or negative. However, like the present study, the Florida study (Polat et al., 2019) noted that the models were only as good as the data used to train them, and that models built using larger, more representative datasets are needed. Since collecting water quality data can be costly, the Florida study (Polat et al., 2019) suggested that a cost-effective way to generate a dataset of sufficient size would be to pool existing datasets from different water sources (e.g., streams, ponds) and regions (e.g., Northeast, Southeast, Southwest). We agree with this recommendation, and think that such multi-regional and multi-year datasets are key to the development of realistic models that can be integrated into on-farm food safety management plans. As mentioned in the statistical sections of the methods or in the discussion below, future studies will need to weigh trade-offs between model interpretability and model accuracy. Despite the aforementioned limitations of the current study and the Florida study (e.g., small sample size, small number of water sources in either the training or test datasets; Polat et al., 2019), these studies suggest that predictive models may be useful for identifying and managing food safety hazards associated with preharvest water use.

### Although, Full Models Built Using Ensemble and “Black-Box” Learners Outperformed Full Models Built Using Interpretable Learners, There Was Not a Single Optimal Learner for Predicting Both *eaeA-stx* and *Salmonella* Presence

The top performing models for both *Salmonella* and *eaeA-stx* were ensemble and/or “black-box” learners (Figure 4). A black-box model is a model that can be viewed in terms of inputs and outputs with limited insights into the internal workings. While some aspects of ensemble learners can be visualized (see Weller et al., 2020a), these models are less interpretable than tree-based (see Bradshaw et al., 2016; Weller et al., 2020b) or regression learners (see Weller et al., 2016), where the exact effect of each feature retained can be estimated and significance assessed. When all data types were used in model development (i.e., the full models) 9 of the 10 top-performing *eaeA-stx* models, and 8 of the 10 top-performing *Salmonella* models were either ensemble (forests, random ferns, or node Harvest) or black-box (instance-based or SVMs) learners. This result was not unexpected, as many ensemble methods were developed to overcome the limitations of interpretable tree-based, rule-based, and regression learners (Breiman, 1996; Li, 2009; Meinshausen, 2010; Özuysal et al., 2010; Li et al., 2011; Kursa, 2014). Indeed, the perennial debate within data science centers on the trade-off between interpretability and performance (Meinshausen, 2010). Since previous papers have outlined these trade-offs we will not focus on them here (e.g., Meinshausen, 2010). However, it is important to note that interpretable learner performance can approach ensemble and “black-box” learners’ performance when (i) there is less noise in the dataset, and (ii) there are strong associations between the outcome and features. Since environmental data (i.e., the data available for use in models to predict foodborne pathogen presence in agricultural water) is inherently noisy due to (i) natural variation in water quality, (ii) the resolution of the spatial data available, and (iii) the imprecision of available weather data (i.e., most farms do not have their own weather stations on-site) future efforts to develop predictive models that can be used to support on-farm decision-making will therefore need to use learners robust to this noise. However, since these models are going to be used by stakeholders, interpretation will also be important. As such, future studies may want to utilize ensemble (e.g., forests) as opposed to “black-box” methods (e.g., SVM, instance-based) since the former can handle noise within the data but are more interpretable than the latter (Table 2).

When we compare performance between the different ensemble models used here there is no clear front runner. In fact, the top-performing *Salmonella* full models were built using different learners than the top-performing *eaeA-stx* full models (Figures 4, 7, 8). This is demonstrative of the *No Free Lunch Theorem* of machine learning, which states that there is not a single, optimal learner that can be applied to all prediction problems (Wolpert and Macready, 1997). Thus, future studies that seek to develop and deploy models on-farms (e.g., to predict the presence of food safety hazards in agricultural water or guide development of water quality sampling plans) should (i) consider model aims and end-user needs (e.g., is accuracy or interpretability more important), and (ii) the explanatory data and computational power available. Moreover, to ensure the best performing model is developed, these future studies should create and compare a handful of models built using different learners and multiple test datasets. Note, that these studies should not use similar, and potentially redundant, learners or develop 23 models per outcome, this was only done in the present, proof-of-concept study to outline the advantages and disadvantages of different machine learners available for model development. Despite the limited overlap between the top-performing *Salmonella* and *eaeA-stx* models, forests were top-ranked learners for both outcomes, and should thus be considered for use in future studies.

Additionally, future studies focused on development of models that can be deployed on-farms should (i) use learners that generate probabilities as opposed to binary labels, (ii) avoid using measures that rely on binary labels during model tuning, training, or selection, and (iii) consider using density plots or split quantile plots as opposed to individual metrics (e.g., Se, Sp) for visualizing model performance in GUI interfaces targeted at end-users. The reasoning behind this recommendation becomes evident when we examine the split-quantile plots for the top-performing *eaeA-stx* model, which was built using the node harvest learner (Figure 8). The split-quantile plot visually depicts how good a model is at categorizing *eaeA*-*stx* positive-samples as positive and *eaeA*-*stx* negative-samples as negative. The split-quantile plot shows that the false positive and false negative rate for the model is substantially lower than the sensitivity (0.89) and specificity (0.52) for the model would suggest. This discrepancy is a due to how sensitivity and specificity are calculated. The output from most learners is the probability of detecting the pathogen in a given sample. To calculate sensitivity and specificity this probability must be dichotomized (i.e., samples classified as either pathogen positive or negative) using a tuned threshold. Whenever a continuous variable is dichotomized information is lost. This is why sensitivity and specificity were not used to rank model performance in the present study. More importantly, the discrepancy between the *eaeA-stx* node harvest model’s split-quantile plot compared to its sensitivity and specificity is illustrative of the fact that model output (i.e., probabilities vs. binary, class labels) can affect perceived model performance, and needs to be considered when selecting models for use in future studies.

### Nested Salmonella Models Built Using Spatial, Physicochemical, and/or Weather Data May Provide a Real-Time, Cost-Effective Tool for Assessing the Food Safety Risks Associated With Preharvest Surface Water Use

**FIGURE 6 |:**
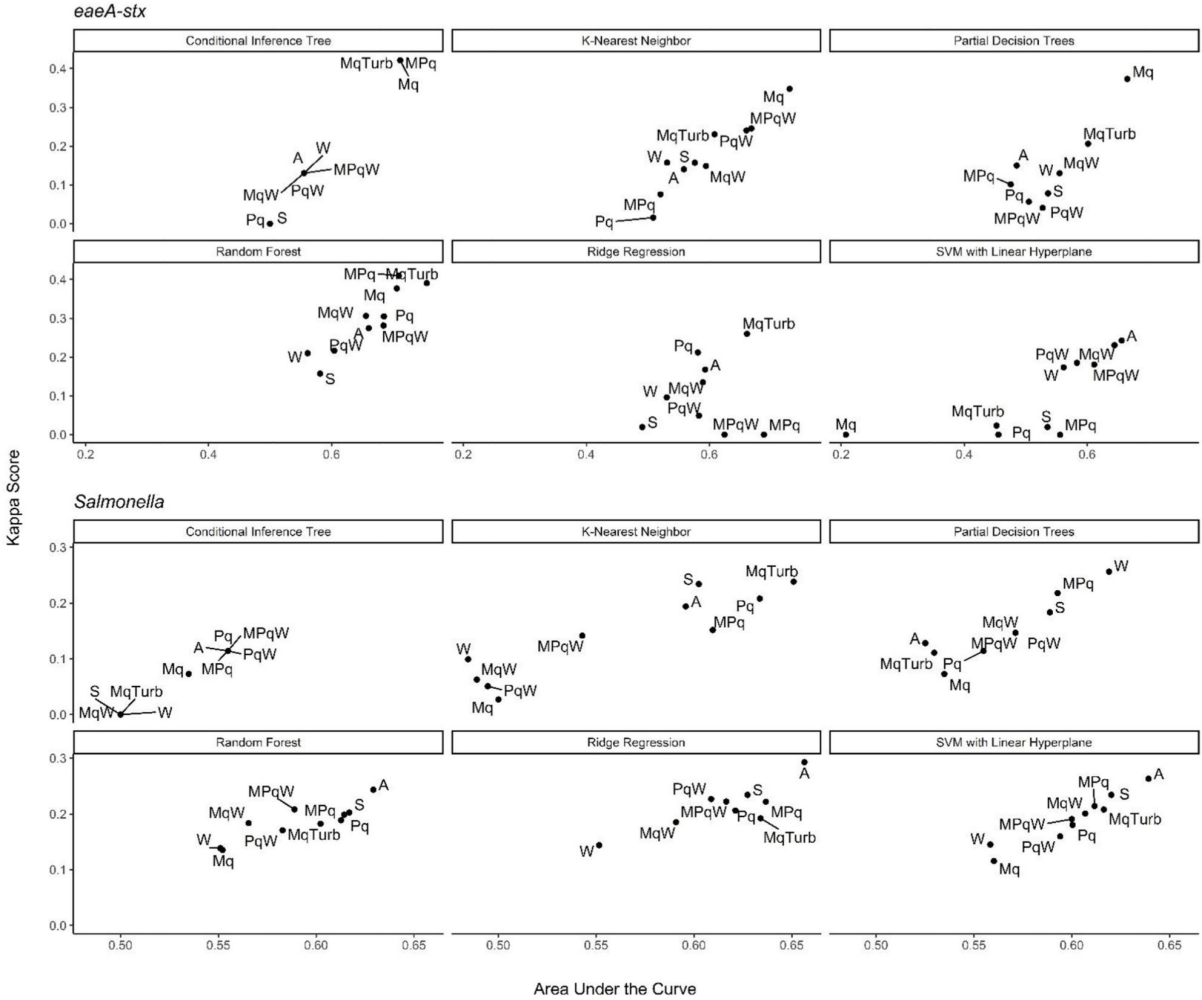
Kappa score and AUC for the nested models. Results are faceted by model outcome and learner: Mq, microbial; MqTurb, microbial data and turbidity; Pq, Physicochemical water quality and air temperature collected on site; W, Weather from publicly-available databases; S, Spatial. With the exception of the Mq models, each nested model used data on site traits (e.g., stream bottom substrate). Top performing models are in the top right corner of each facet.

**FIGURE 7 |:**
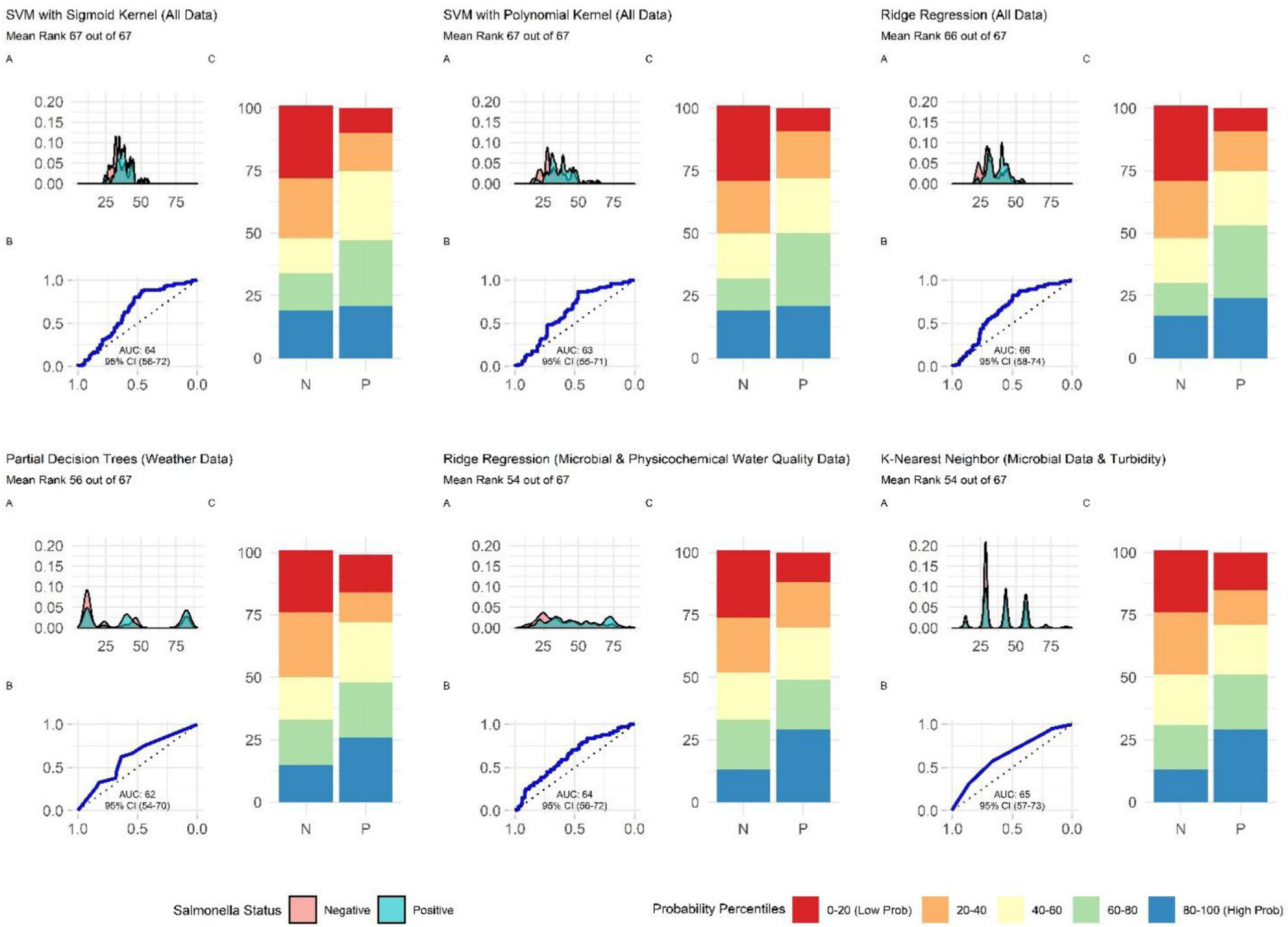
Plots showing the performance of the top-ranked full and nested *Salmonella* models. **(A)** Shows how well the model can distinguish samples that tested positive and negative for *Salmonella*. The x-axis of **(A)** is the probability of *Salmonella* detection generated by the model, and the y-axis is density. **(B)** Is the receiver-operating curve (ROC) for the model; the x-axis is 1-Specificity and the y-axis is Sensitivity. **(C)** Shows how well the model is at accurately classifying positive and negative samples. The split quantiles plot is generated by sorting the test data from lowest to highest probability of *Salmonella* detection based on the given model. The test data is then divided into quantiles (based on the percentile the probability falls into). The proportion of samples in each quantile that were actually *Salmonella*-positive or negative were then plotted. A good model would identify all low probability percentile samples (red) as negative (N) and all high probability percentile samples (blue) as positive (P).

In addition, to the full *Salmonella* models, we also built a series of nested models using between one and three feature types (Figure 6; Supplementary Figure 3). All nested models performed worse than the top-nine full models (Supplementary Table 2). The top-performing *Salmonella* nested models were the partial decision tree (PART) built using weather features, and the ridge regression built using microbial and physicochemical water quality features (Figure 7).

Interestingly, while none of the nested models built using spatial data were among the top-three nested *Salmonella* models, all spatial models consistently performed well-compared to nested models that excluded spatial data (Figure 6). In fact, 4 of the top 10 nested models were built using just spatial data (Figure 6; Supplementary Table 2). Five models built using physicochemical water quality features were also in the top 10 nested learners, however, these models were often also built using both weather and physicochemical features (Figure 6; Supplementary Table 2). This is consistent with the findings of Polat et al. (2019) who examined the ability of various models to predict *Salmonella* presence in Central Florida irrigation ponds. Polat et al. (2019) found that accurate models (>70% classification accuracy on training data) could be achieved using only one or two water quality or weather features. The strong performance of the spatial, and physicochemical water quality and weather models in the study reported here, and of similar models in Polat et al. (2019) suggests that accurate models for predicting *Salmonella* presence in agricultural water can be developed (i) using only one or two feature types, and (ii) without using microbial data. This is a substantial finding since costs associated with microbial water quality testing have been identified as a key concern among growers and it can take >24 h to get *E. coli*-based water quality test results (Astill et al., 2018; Wall et al., 2019). This 24 h lag, as well as the well-documented spatiotemporal variability in microbial water quality, means *E. coli*-based test results may not be associated with the presence of food safety hazards in water at the time of water use (Havelaar et al., 2017; Weller et al., 2020a). As such, models that use physicochemical, weather and/or spatial but not microbial data to predict when and where *Salmonella* is likely to be present in agricultural water may provide a real-time, cost-effective tool for managing the food safety risks associated with the use of surface water contaminated by *Salmonella* for produce production. Such a tool could be used as an alternative or supplement to existing *E. coli* water quality testing programs.

### Nested *eaeA-stx* Models Built Using Learners That Employ a Divide-and-Conquer Algorithm and Microbial Features Outperformed Models That Were Built Using Other Learners and Feature Types

**FIGURE 8 |:**
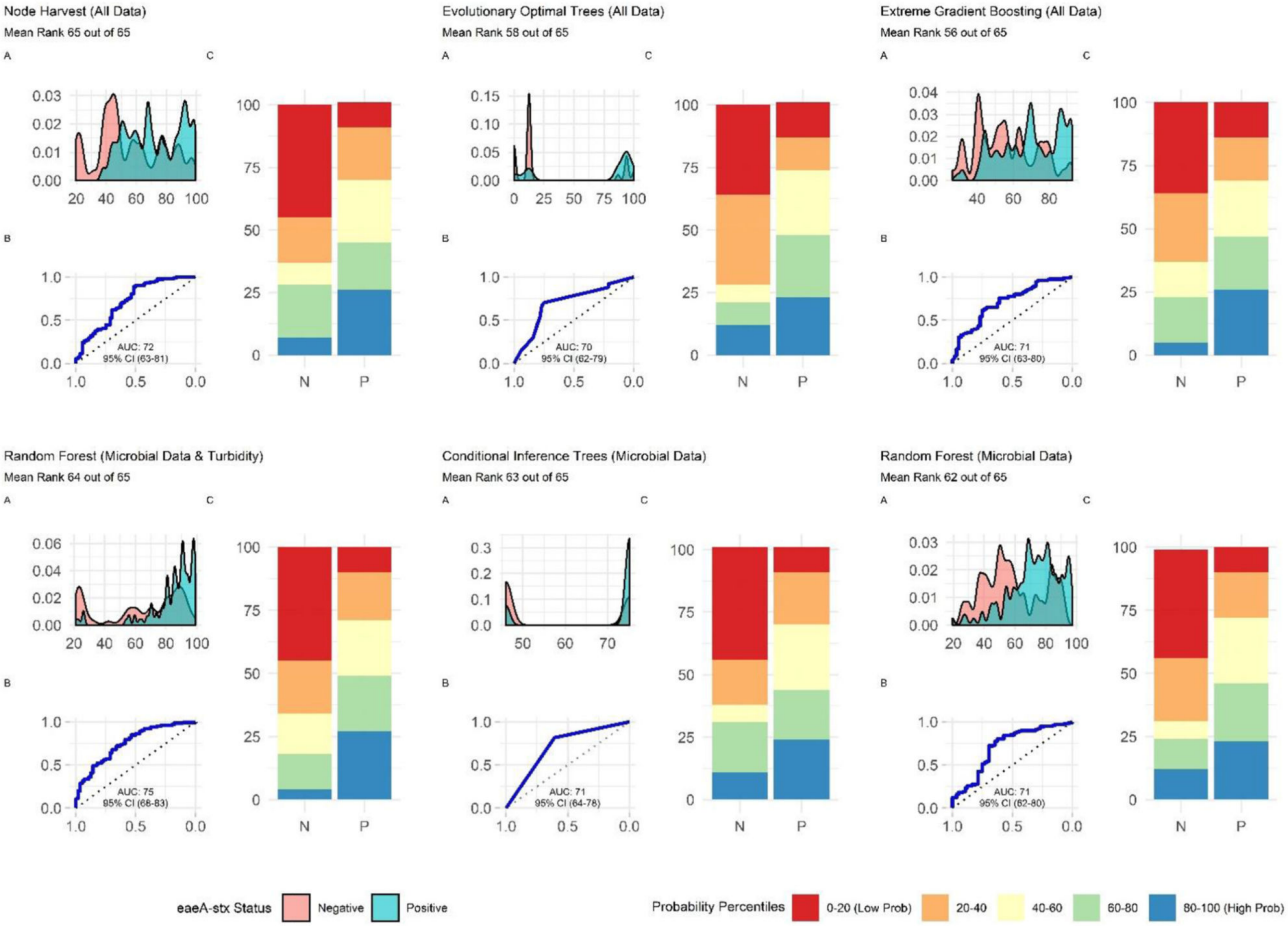
Plots showing the performance of the top-ranked full and nested *eaeA-stx* models. **(A)** Shows how well the model can distinguish samples that tested positive and negative for *eaeA-stx*. The x-axis of (A) is the probability of *eaeA-stx* detection generated by the model, and the y-axis is density. **(B)** Is the receiver-operating curve (ROC) for the model; the x-axis is 1-Specificity and the y-axis is Sensitivity. **(C)** Shows how well the model is at accurately classifying positive and negative samples. The split quantiles plot is generated by sorting the test data from lowest to highest probability of *eaeA-stx* detection based on the given model. The test data is then divided into quantiles (based on the percentile the probability falls into). The proportion of samples in each quantile that were actually *eaeA-stx* -positive or negative were plotted. A good model would identify all low probability percentile samples (red) as negative (N) and all high probability percentile samples (blue) as positive (P).

The top-performing nested *eaeA-stx* models were the RF model built using microbial and turbidity data, and the cTree model built using microbial data (Figure 8). It is important to note that the models built using the cTree algorithm and (i) microbial data, (ii) microbial and turbidity data, and (iii) microbial and physicochemical water quality data, were exactly the same and included a single split based on *E. coli* levels. These three nested models were therefore treated as the same model when ranking models based on performance (Supplementary Table 3; Figure 8). While all nested models performed worse than the top-ranked full model (i.e., node harvest), the RF model built using microbial and turbidity was the second-best model in the study reported here (Supplementary Table 3). All *eaeA-stx* models that included microbial data outperformed models built using the same learner but without microbial data. This strongly suggests that microbial data, and thus generic *E. coli*-based water quality monitoring, are useful tools for predicting when and where pathogenic *E. coli* may be present in New York agricultural water. Although additional research is needed to test this conclusion for waterways outside the study region, this finding is consistent with past studies that reported strong associations between detection of pathogenic *E. coli* (or pathogenic *E. coli* markers, such as *eaeA* and *stx*), and generic *E. coli* levels (Bradshaw et al., 2016; Weller et al., 2020a). For example, Bradshaw et al. (2016) used CART learners to predict *stx* presence in Georgia waterways, and found that *E. coli* was able to accurately identify *stx*-positive samples when air temperature ≥13°C. Interestingly, the best nested *eaeA-stx* models included *E. coli* levels, turbidity levels, and site characteristics. This supports the conclusion of previous studies (McEgan et al., 2013a; Bradshaw et al., 2016; Weller et al., 2020a) that the relationship between *E. coli* levels and pathogen presence is modified by environmental context at time of sampling (or water use). These environmental modifiers (e.g., turbidity and site-characteristics) may be appropriate as supplemental indicators of potential pathogen presence; indeed, several past studies have suggested this (McEgan et al., 2013a; Bradshaw et al., 2016; Polat et al., 2019). Unfortunately, the aforementioned limitations of *E. coli*-based models (costs associated with microbial testing, and the potential disconnect between *E. coli* levels at time of sample collection and time of water use), may complicate the development and deployment of predictive model-based decision-support tools that incorporate *E. coli* data.

## CONCLUSION

This was a proof-of-concept study designed to provide guidance on how predictive models (e.g., different machine learners and feature types that should be the foci of future model-development efforts) can be incorporated into on-farm decision-support tools. For example, we highlight that inclusion of microbial features were key to developing accurate models to predict pathogenic *E. coli* presence but not *Salmonella* presence in New York streams used to source water for produce production. As part of this discussion, the current study also highlighted the advantages and disadvantages associated with each learner, and the importance of considering the trade-offs between model interpretability, and accuracy. In addition to identifying learners and data types that should be the focus of future studies, this study also sought to determine if machine learning-based models are likely to be useful for managing *Salmonella* and pathogenic *E. coli* risks associated with preharvest water use. Based on the findings of this, and the only other study (Polat et al., 2019), to the author’s knowledge, that used machine learners to predict enteric pathogen presence in irrigation water, predictive models may be useful for identifying when and where pathogens are likely to be present in agricultural water. Although predictive models could be an alternative to *E. coli* water quality testing, they could also be used to supplement ongoing *E. coli*-based water quality monitoring (e.g., to target sampling to sites and times with a higher risk of pathogen presence). Given the importance of microbial features to the development of accurate models for predicting *eaeA*-*stx* presence, predictive models should specifically be considered as a supplementary, rather than an alternative, approach for managing pathogenic *E. coli* contamination of agricultural water sources.

## Supplementary Material

Suppl

## Figures and Tables

**TABLE 1 |:** Foodborne pathogen prevalence and *E. coli* levels in New York streams used for produce production.

Year	No. of		Prevalence (No. of positive samples)			Median MPN of *E. coli*/100-mL (Min.–Max.)
	Streams	Samples	Culture-confirmed	PCR-screen positive		
			*Salmonella*	*eaeA*	*stx*^a^	

2017	6	181	44% (80)	94% (171)	69% (125)	160.4 (18.5–>2,419.6)
2018	68	191	41% (79)	99% (190)	70% (133)	211.4 (2.0–>2,419.6)
Total	68	372	43% (159)	97% (361)	69% (258)	193.5 (2.0–>2,419.6)

a The outcome of the eaeA-stx models was codetection of both the eaeA and the stx genes; in both years all stx-positive samples were also eaeA-positive, as a result the prevalence of samples that were positive for both genes was 69% in 2017 and 68% in 2018.

**TABLE 2 |:** List of learners used here, including advantages and disadvantages of each learner as implemented in the R package used here^a^.

Learners	Package	*n < p*	Centering and scaling needed	In features it can handle				Automatic feature selection	Interpretable
				Correlation	Missingness	Near-Zero	Noise^b^		

Bayesian Learners									
Naive Bayes	e1071 (Meyer et al., 2019)	**Y**	**N**	•	**Y**	**N**	•	**N**	**N**
Tree-Based Learners									
Classification tree^b^	rpart (Therneau and Atkinson, 2019)	**Y**	**N**	•	**Y**	**Y**	•	**Y**	**Y**
Conditional tree	party (Zeileis et al., 2008)	**Y**	**N**	**Y**	**Y**	**Y**	•	**Y**	**Y**
Evolutionary optimal tree	evtree (Grubinger et al., 2014)	**Y**	**N**		**N**	**Y**	•	**Y**	**Y**
Ensemble Learners^b^									
Conditional forest	party (Zeileis et al., 2008)	**Y**	**N**	**Y**	•	**Y**	**Y**	•	•
Node harvest^c^	nodeHarvest (Meinshausen, 2015)	**Y**	**N**	•	**Y**	**Y**	**Y**	**Y**	•
Random forest^c^	randomForest (Liaw and Wiener, 2002)	**Y**	**N**	•	**Y**	**Y**	**Y**	•	•
Regularized RF	RRF (Deng, 2013; Deng and Runger, 2013)	**Y**	**N**	**Y**	**N**	**Y**	**Y**	**Y**	•
Random ferns^d^	rferns (Kursa, 2014)	•	**Y**	**Y**	**N**	**N**	**Y**	**N**	•
Random KNN^d,e^	rknn (Li, 2015)	•	**Y**	**Y**	**N**	**N**	•	**N**	**N**
Extreme gradient boosting	xgboost (Chen et al., 2020)	**Y**	**N**	**Y**	**Y**	**Y**	**Y**	•	•
Instance-Based Learners^e^									
k-Nearest neighbor	kknn (Schliep and Hechenbichler, 2016)	•	**Y**	**N**	**N**	**N**	•	**N**	**N**
Weighted kKNN	kknn (Schliep and Hechenbichler, 2016)	•	**Y**	**N**	**N**	**N**	•	**N**	**N**
Penalized Regression									
Elastic net	glmnet (Friedman et al., 2010)	**Y**	**Y**	**Y**	**N**	**N**	**N**	**Y**	**Y**
Lasso	glmnet (Friedman et al., 2010)	**Y**	**Y**	**Y**	**N**	**N**	**N**	**Y**	**Y**
Ridge	glmnet (Friedman et al., 2010)	**N**	**Y**	**Y**	**N**	**N**	**N**	**N**	**Y**
Rule-Based Learners									
JRip	RWeka (Hornik et al., 2009)	**Y**	**N**		**Y**			**Y**	**Y**
One rule	RWeka (Hornik et al., 2009)	**Y**	**N**	**Y**	**Y**			**Y**	**Y**
Partial decision lists	RWeka (Hornik et al., 2009)	**Y**	**N**	**Y**	**Y**	**Y**	**Y**	**Y**	**Y**
SVM	e1071 (Meyer et al., 2019)	**Y**	**Y**	•	**N**	**Y**	**N**	**N**	**N**

This table was adapted from Kuhn and Johnson (2016) to include all learners used here. The information reported here is based on the papers cited for each learner in the methods section, and the constraints of the R packages used to implement the learners in this study (based on the version available in January 2020). **Y** means the learner meets the condition in the header. **N** means the learner does not meet this conditional. • = the learner is in between (e.g., random forest is not as interpretable as tree-based methods but is not a 100% black-box method like support vector machines). If the cell is blank it means there was limited information on this parameter for the given learner.

b It is important to note that although tree-based methods are relatively robust to noise in the features, they are less robust than tree-based ensembles. Theoretically, ensemble methods are more robust to noise in the features than constituent models used to build the ensemble (rFERNS should be more robust than Naïve Bayes, rKNN should be more robust then wKNN and kKNN, forests should be more robust than trees).

c Preferentially selects continuous variables and categorical variables with many levels as the splitting variable resulting in variable selection bias (Strobl et al., 2007, 2008, 2009). Conditional inference trees and conditional forests were developed to overcome these limitations (Strobl et al., 2007, 2008, 2009).

d Predicts class labels but not probability of detecting a positive.

e Feature selection recommended prior to model development.

## Data Availability

The data analyzed in this study is subject to the following licenses/restrictions: confidential geo-referenced data was used. De-identified data is available upon request from the study authors; select data are available at foodmicrobetracker.com. Requests to access these datasets should be directed to wellerd2@gmail.com; dlw263@cornell.edu.

## Footnotes

1 Machine Learning in R [R package mlr version 2.15.0].
