# Supplementary material for: Predictive Models May Complement or Provide an Alternative to Existing Strategies for Assessing the Enteric Pathogen Contamination Status of Northeastern Streams Used to Provide Water for Produce Production
Source: Front Sustain Food Syst. Author manuscript; Available in PMC 2021 Mar 30. (PMC8009603; doi:10.3389/fsufs.2020.561517)
Supplement: Suppl [file NIHMS1680359-supplement-Suppl.docx]

Figure S1: Plot comparing performance measures for predictions made using the training and predictions made using the test data. Higher values for the training data compared to the test data indicates overfitting. Note, not all learners are shown for all measures. When a learner is missing for a given measure this indicates that the learner performed poorly on one of the datasets (i.e., called all samples positive or all samples negative for the target), or that the given measure could not be calculate for that learner. For baseline models, the letters refer to the organism the cutoff is based on (EC= *E. coli,* TC= total coliforms), and the number refers to the cut-off value (e.g., EC.126 is based on a cut-off of 126 MPN of *E. coli*/100-mL).


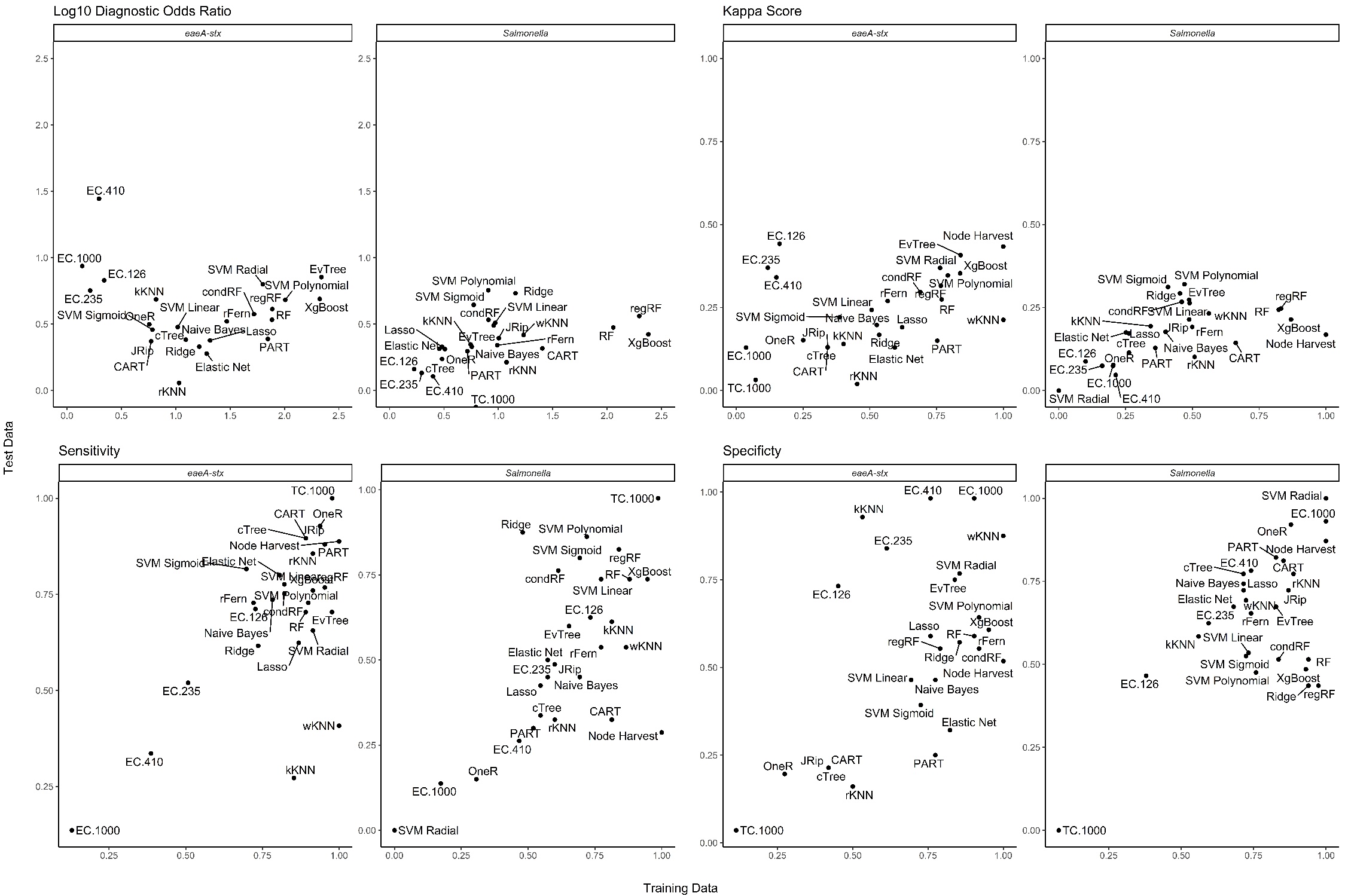


Figure S2: Plot showing sensitivity versus 1-specificity for the full and baseline models. Top performing models are in the top right corner each facet. For baseline models, the letters refer to the organism the cutoff is based on (EC= *E. coli,* TC= total coliforms), and the number refers to the cut-off value (e.g., EC.126 is based on a cut-off of 126 MPN of *E. coli*/100-mL). Top performing models are closest to the top left corner.


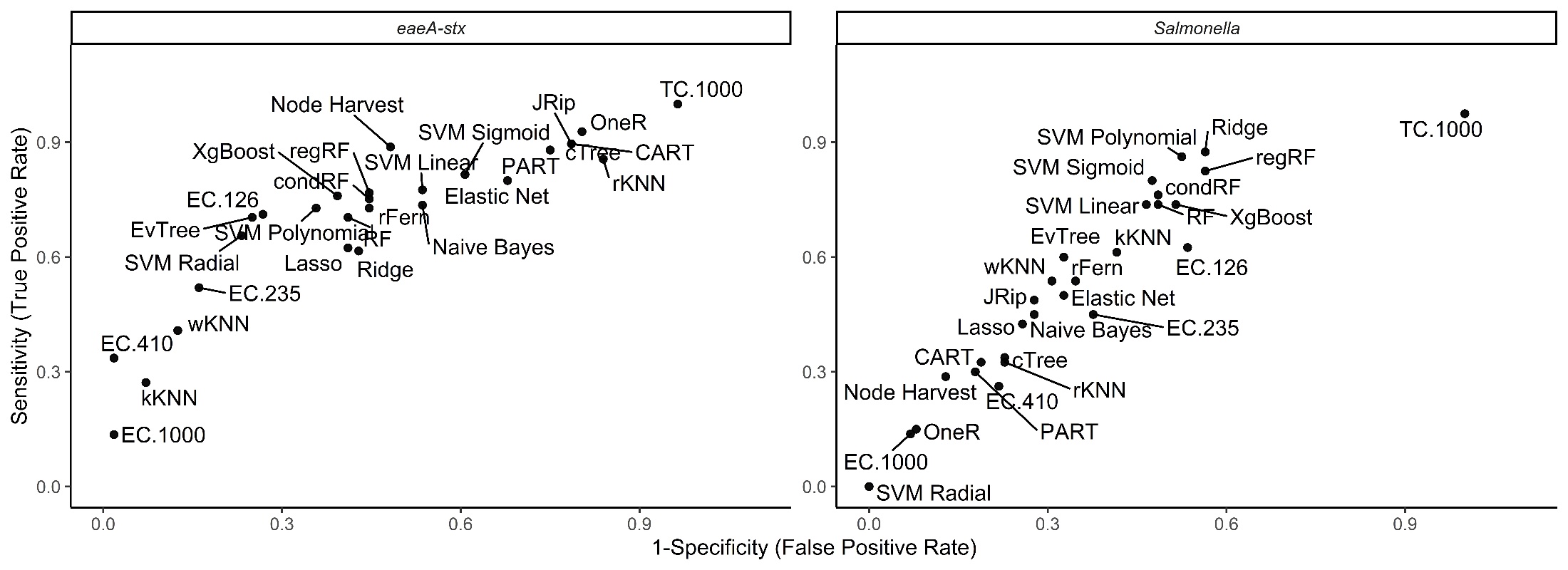


Figure S3: Plot showing sensitivity and 1-specificity for the nested models. Results are faceted by model outcome and learner: Mq = microbial, MqTurb = microbial data and turbidity, Pq= Physicochemical water quality and air temperature collected on site; W= Weather from publicly-available databases; S = Spatial. With the exception of Mq models, each model included data on site traits (e.g., stream bottom substrate). Top performing models are in the top left corner of each facet.


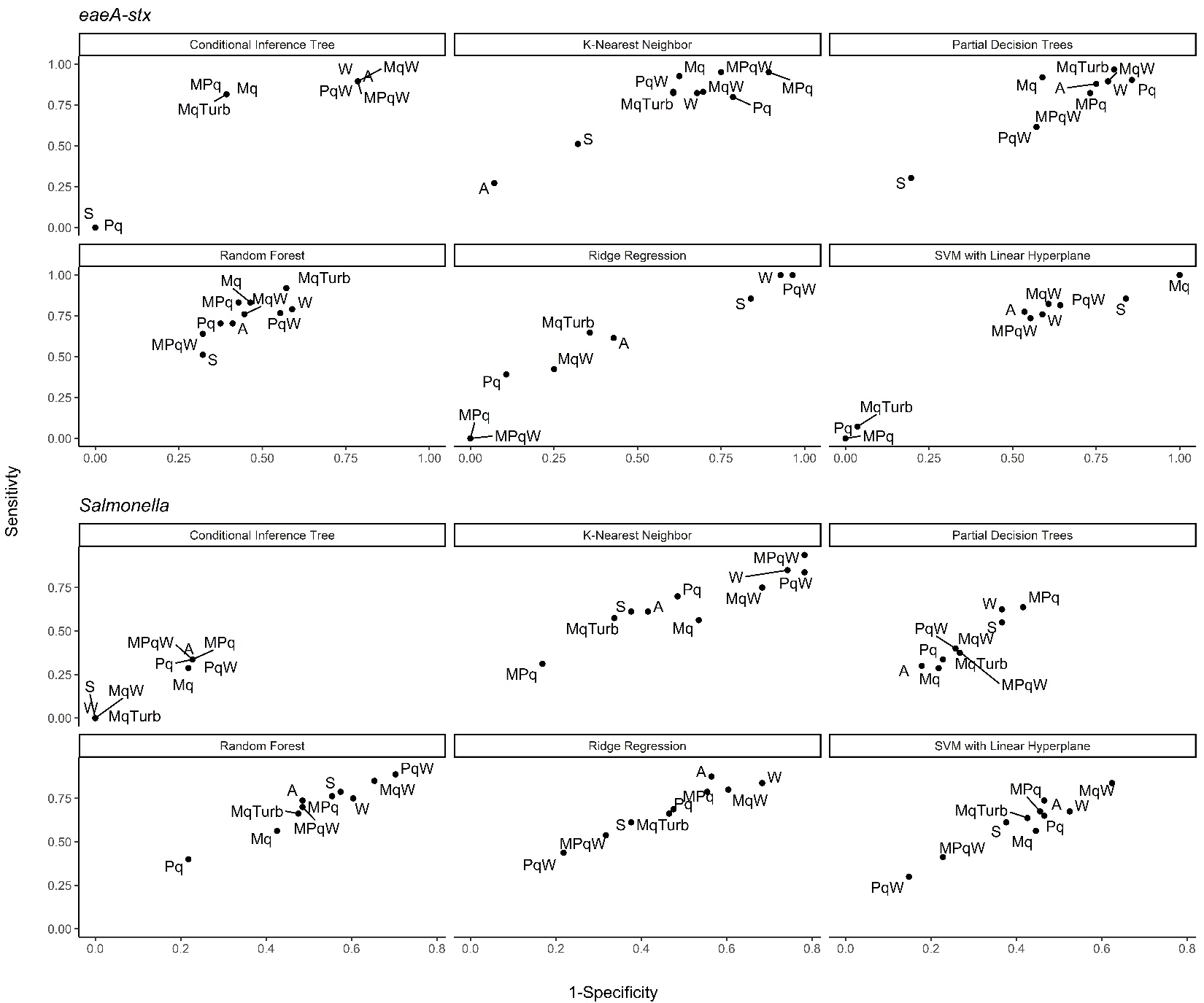


Table S1: Factors included in the analyses reported here. Values for all weather factors with the exception of temperature were calculated for 0-1, 1-2, 2-3, 3-4, 4-5, 5-10, 10-20 and 20-30 d before sample collection (BSC). Values for temperature were calculated for 0-5, 5-10, 10-20 and 20-30 d BSC due to the strong correlation between temperature 0-1, 1-2, 2-3, 3-4 and 4-5 d BSC.

| Factor | | | | Description | Data Type ^a^ | Date | Citations and Websites |
| --- | --- | --- | --- | --- | --- | --- | --- |
| Data Extracted from Publicly-Available Databases | | | | |  |  |  |
|  | Culverts: Carrying a waterway under a state highway and have a span of 5 to 20 feet | | | |  | 2014 | (1–3) |
|  |  | Present | | Were culverts present upstream? | S |  |  |
|  |  | Density | | Upstream density (no. of per 10 km^2^) | S |  |  |
|  | Dams | | |  |  | 2018 | (4) |
|  |  | Density | | Upstream density (no. of per 10 km^2^) | S |  |  |
|  | Road Crossing | | |  |  | 2015 | (5) |
|  |  | | Density | Upstream density (no. of per 10 km^2^) | S |  |  |
|  |  | | Min. Dist. | The flow path distance to nearest point upstream where a road crossed the stream (km). | S |  |  |
|  | Outfalls: Municipal stormwater outfalls along or near highways (presence is an indicator of urbanization) | | | |  | 2008 | (6–8) |
|  |  | Present | | Were stormwater outfalls present upstream? | S |  |  |
|  |  | Density | | Upstream density (no. of per 10 km^2^) | S |  |  |
|  | Municipal Wastewater Discharge Sites: Based on permits issued under the NYS^a^ Pollutant Discharge Elimination System (SPDES) | | | |  |  |  |
|  |  | Present | | Were wastewater discharge sites present upstream? | S |  |  |
|  |  | Density | | Upstream density (no. of per 10 km^2^) | S |  |  |
|  | Industrial Wastewater Discharge Sites: Based on permits issued under the NYS^b^ Pollutant Discharge Elimination System (SPDES) | | | |  | 2018 | (9) |
|  |  | Present | | Were wastewater discharge sites present upstream? | S |  |  |
|  |  | Density | | Upstream density (no. of per 10 km^2^) | S |  |  |
|  | In-stream Waterbodies: Bodies of water within the stream channel (e.g., mill ponds, impoundments, lakes) | | | |  | 2017 | (10) |
|  |  | Present | | Were waterbodies upstream? | S |  |  |
|  |  | Density | | Upstream density (no. of per 10 km^2^) | S |  |  |
|  | Septic System Density | | | Upstream density (no. per 10 km^2^). | S | 2011 | (11) |
|  | Solid Waste Site: Based on permits issued by NYS that allow application and spreading of manure, human septage, food processing, or other waste | | | |  | 2019 | (12) |
|  |  | Density | | Upstream density (no. of per 10 km^2^) | S |  |  |
|  | Land Cover ^c^ | | | |  | 2016 | (13, 14) |
|  |  | Open Water | | Class 11 in National Land Cover Database (NLCD) | S |  |  |
|  |  | Cropland | | Cropland; Class 82 in NLCD | S |  |  |
|  |  | Pasture | | Pasture; Class 81 in NLCD | S |  |  |
|  |  | Developed | | Developed; Classes 21-24 in NLCD | S |  |  |
|  |  | For-Wet | | Natural cover; Classes 41-43, 51-52, 90, and 95 in NLCD (i.e., forest, shrubland or wetland) | S |  |  |
|  |  | Impervious | | Percent of upstream watershed that was under impervious cover | S |  |  |
|  | Watershed Area | | | Total area of upstream watershed (10-km^2^) | S | - | - |
|  | Stream Stats Data | | |  |  | 2019 | [streamstats.usgs.gov/ss/](https://streamstats.usgs.gov/ss/) |
|  |  | BSLOPCM | | Mean basin lope determined by summing lengths of all contours in basin, multiplying by contour interval, and dividing product by drainage area | S |  |  |
|  |  | CONTOUR | | Total length of all elevation contours in drainage area in miles | S |  |  |
|  |  | CSL10_85 | | Change in elevation divided by length between points 10 and 85 percent of distance along main channel to basin divide | S |  |  |
|  |  | CSL1085LO | | Change in elevation between points 10 and 85 percent of length along the lower half of the main flow path divided by length between the points | S |  |  |
|  |  | CSL1085UP | | Change in elevation between points 10 and 85 percent of length along the upper half of the main flow path divided by length between the points | S |  |  |
|  |  | EL1200 | | Percentage of basin at or above 1200 ft elevation | S |  |  |
|  |  | LAGFACTOR | | Basin Lag factor as defined in SIR 2006-5112 | S |  |  |
|  |  | Length | | Length along the main channel from the measuring location extended to the basin divide | S |  |  |
|  |  | SLOPERAT | | Ratio of main channel slope to basin slope | S |  |  |
|  |  | SSURGOA | | Percentage of area of Hydrologic Soils Type A from SSURGO | S |  |  |
|  |  | SSURGOB | | Percentage of area of Hydrologic Soils Type B from SSURGO | S |  |  |
|  |  | Storage | | Percentage of area of storage (lake, ponds, reservoirs, wetlands) | S |  |  |
|  |  | StreamLevel | | Stream level | S |  |  |
|  |  | StreamOrder | | Strahler stream order | S |  |  |
|  |  | ArbolateSu | | An estimate of miles of stream upstream of a flowline | S |  |  |
| Water Quality and Hydrological Conditions at Time of Sample Collection | | | | |  |  |  |
|  | Air Temp. at site | | | Air temperature measured at the sampling site at the time of sample collection (°C) | Pq | - | - |
|  | *E. coli ^d^* | | | Log_10_ *E. coli* concentration in the waterway (MPN/100 mL) | Mq | - | - |
|  | Conductivity | | | Conductivity (Log_10_ uS/cm) | Pq | - | - |
|  | Dissolved oxygen | | | Dissolved oxygen levels (mg/L) | Pq | - | - |
|  | Flow rate | | | Flow rate measured 3-6” below the surface (m/s) | Pq | - | - |
|  | pH | | | pH | Pq | - | - |
|  | Turbidity | | | Turbidity (Log_10_ NTU) | Pq | - | - |
|  | Water Temp. | | | Water temperature (°C) | Pq | - | - |
| Field-Collected Site Data | | | | |  |  |  |
|  | Ditch | | | Did a roadside ditch intersect the stream < 20 m upstream of the sample site? | FC | ~~-~~ | ~~-~~ |
|  | Bottom Substrate: Composition of the stream bottom in the reach 10 m upstream of the sample site. The different categories of substrate were boulder, bedrock, cobble or larger, coarse gravel, fine gravel, sand, clay and organic matter. | | | |  | ~~-~~ | (15) |
|  |  | Rocky | | Was the substrate that comprised the majority of the bottom rocky (bedrock, boulder, cobble, or gravel) or not rocky (sand, clay, or organic matter/silt)? | FC | - | - |
|  |  | Sand | | Was sand present along the stream bottom? | FC | - | - |
|  |  | Clay | | Was clay present along the stream bottom? | FC | - | - |
|  |  | Organic Matter | | Was organic matter present along the stream bottom? | FC | - | - |
|  |  | Cobble or Larger | | Were cobble, boulders or bedrock along the stream bottom? | FC | - | - |
|  |  | Fine gravel | | Was fine gravel present along the stream bottom? | FC | - | - |
|  |  | Coarse gravel | | Was coarse gravel present along the stream bottom? | FC | - | - |
| Weather | | | |  |  |  |  |
|  | Avg. Air Temp. | | | Average temperature (°C) either 0-5, 5-10, 10-20 or 20-30 d before sample collection | W | - | [newa.cornell.edu](http://newa.cornell.edu/) |
|  | Avg. Solar Radiation | | | Average solar radiation (MJ/m^2^) either 0-1,1-2, 2-3, 3-4, 4-5, 5-10, 10-20 or 20-30 d before sample collection | W | - | [newa.cornell.edu](http://newa.cornell.edu/) |
|  | Total rainfall | | | Total rainfall (cm) either 0-1,1-2, 2-3, 3-4, 4-5, 5-10, 10-20 or 20-30 d before sample collection | W | - | [newa.cornell.edu](http://newa.cornell.edu/) |

^a^To assess the relative information gain associated with using different data types to build predictive models, two sets of analyses were performed. In the first set of analyses, each learner and the full set of factors listed in Table S1 were used to develop 23 predictive models per outcome. In the second set of analyses, the factors listed in Table S1 were divided into four groups: microbial data; physicochemical water quality and temperature data collected on site; weather data obtained from NEWA weather stations; and spatial data. Nested models were then built using different combinations of these 4 data types: Mq = microbial, MqTurb = microbial data and turbidity, Pq= Physicochemical water quality and air temperature collected on site; W= Weather from publicly-available databases; S = Spatial. With the exception of models built using just the microbial data, all models included field-collected site traits (FC).

^b^ New York State = NYS

^c^ For each land cover class we calculated the proportion of (i) the total watershed area, (ii) the stream corridor (i.e., area 0-60 m from the stream corridor), (iii) the flood plain (based on shapefile downloaded from NYS Department of Environmental Conservation), and (iv) the area immediately upstream (0-100 m) of the sampling site.

^d^ Limit of detection = LOD; the upper limit of detection for the *E. coli* and total coliforms assay was 2,419.6 MPN/100-mL.

Table S2: Performance measures for each model where *Salmonella* isolation was the outcome.

| **Learner** | | **Data Types^a^** | **Mean Rank** | **AUC^b^** | **DOR^c^** | **Informedness^d^** | **Kappa** | **PLR^e^** | **NLR^f^** | **Markedness^g^** | **Se^h^** | **Sp^i^** |
| --- | --- | --- | --- | --- | --- | --- | --- | --- | --- | --- | --- | --- |
| Bayesian Learners | |  |  |  |  |  |  |  |  |  |  |  |
|  | Naive Bayes | All | 39 | 0.63 | 2.13 | 0.17 | 0.18 | 1.62 | 0.76 | 0.18 | 0.45 | 0.72 |
| Baseline Models | |  |  |  |  |  |  |  |  |  |  |  |
|  | *E. coli* |  |  |  |  |  |  |  |  |  |  |  |
|  | 1,000 CFU/100-mL (EC.1000) | - | 32 | NA | 2.14 | 0.07 | 0.07 | 1.98 | 0.93 | 0.19 | 0.14 | 0.93 |
|  | 126 CFU/100-mL (EC.126) | - | 26 | NA | 1.45 | 0.09 | 0.09 | 1.17 | 0.81 | 0.09 | 0.63 | 0.47 |
|  | 235 CFU/100-mL (EC.235) | - | 21 | NA | 1.36 | 0.07 | 0.07 | 1.20 | 0.88 | 0.08 | 0.45 | 0.62 |
|  | 410 CFU/100-mL (EC.410) | - | 16 | NA | 1.28 | 0.04 | 0.05 | 1.21 | 0.94 | 0.06 | 0.26 | 0.78 |
|  | Total Coliforms |  |  |  |  |  |  |  |  |  |  |  |
|  | 1,000 CFU/100-mL (TC.1000) | - | 34 | NA | 0.00 | -0.03 | -0.02 | 0.98 | NA | -0.56 | 0.98 | 0.00 |
| Tree-based Learners | |  |  |  |  |  |  |  |  |  |  |  |
|  | Classification Tree (CART) | All | 28 | 0.54 | 2.08 | 0.14 | 0.14 | 1.73 | 0.83 | 0.18 | 0.33 | 0.81 |
|  | Conditional Inference Tree (CTree) |  |  |  |  |  |  |  |  |  |  |  |
|  |  | All | 19 | 0.55 | 1.73 | 0.11 | 0.11 | 1.48 | 0.86 | 0.14 | 0.34 | 0.77 |
|  |  | Mq | 9 | 0.53 | 1.45 | 0.07 | 0.07 | 1.32 | 0.91 | 0.09 | 0.29 | 0.78 |
|  |  | MqTurb | 38 | 0.50 | NA | 0.00 | 0.00 | NA | 1.00 | NA | 0.00 | 1.00 |
|  |  | MPq | 20 | 0.55 | 1.73 | 0.11 | 0.11 | 1.48 | 0.86 | 0.14 | 0.34 | 0.77 |
|  |  | MPqW | 20 | 0.55 | 1.73 | 0.11 | 0.11 | 1.48 | 0.86 | 0.14 | 0.34 | 0.77 |
|  |  | MqW | 39 | 0.50 | NA | 0.00 | 0.00 | NA | 1.00 | NA | 0.00 | 1.00 |
|  |  | Pq | 20 | 0.55 | 1.73 | 0.11 | 0.11 | 1.48 | 0.86 | 0.14 | 0.34 | 0.77 |
|  |  | PqW | 20 | 0.55 | 1.73 | 0.11 | 0.11 | 1.48 | 0.86 | 0.14 | 0.34 | 0.77 |
|  |  | S | 38 | 0.50 | NA | 0.00 | 0.00 | NA | 1.00 | NA | 0.00 | 1.00 |
|  |  | W | 39 | 0.50 | NA | 0.00 | 0.00 | NA | 1.00 | NA | 0.00 | 1.00 |
|  | Evolutionary Optimal Tree (EvTree) | All | 58 | 0.62 | 3.09 | 0.27 | 0.27 | 1.84 | 0.59 | 0.27 | 0.60 | 0.67 |
| Ensemble Learners | |  |  |  |  |  |  |  |  |  |  |  |
|  | Conditional Forest (condRF) | All | 63 | 0.64 | 3.41 | 0.28 | 0.27 | 1.57 | 0.46 | 0.28 | 0.76 | 0.51 |
|  | Node Harvest | All | 34 | 0.54 | 2.73 | 0.16 | 0.17 | 2.23 | 0.82 | 0.25 | 0.29 | 0.87 |
|  | Random Forest (RF) |  |  |  |  |  |  |  |  |  |  |  |
|  |  | All | 57 | 0.63 | 2.98 | 0.25 | 0.24 | 1.52 | 0.51 | 0.26 | 0.74 | 0.51 |
|  |  | Mq | 23 | 0.55 | 1.73 | 0.14 | 0.14 | 1.32 | 0.76 | 0.13 | 0.56 | 0.57 |
|  |  | MqTurb | 36 | 0.60 | 2.17 | 0.19 | 0.18 | 1.39 | 0.64 | 0.18 | 0.66 | 0.52 |
|  |  | MPq | 46 | 0.61 | 2.58 | 0.21 | 0.20 | 1.38 | 0.53 | 0.22 | 0.76 | 0.45 |
|  |  | MPqW | 44 | 0.59 | 2.48 | 0.21 | 0.21 | 1.44 | 0.58 | 0.21 | 0.70 | 0.51 |
|  |  | MqW | 45 | 0.57 | 3.01 | 0.20 | 0.18 | 1.30 | 0.43 | 0.25 | 0.85 | 0.35 |
|  |  | Pq | 41 | 0.61 | 2.39 | 0.18 | 0.19 | 1.84 | 0.77 | 0.21 | 0.40 | 0.78 |
|  |  | PqW | 46 | 0.58 | 3.33 | 0.18 | 0.17 | 1.26 | 0.38 | 0.27 | 0.89 | 0.30 |
|  |  | S | 49 | 0.62 | 2.75 | 0.21 | 0.20 | 1.37 | 0.50 | 0.24 | 0.79 | 0.43 |
|  |  | W | 28 | 0.55 | 1.97 | 0.15 | 0.14 | 1.24 | 0.63 | 0.17 | 0.75 | 0.40 |
|  | Regularized RF (regRF) | All | 60 | 0.64 | 3.64 | 0.26 | 0.25 | 1.46 | 0.40 | 0.30 | 0.83 | 0.44 |
|  | Random Ferns (rFern) | All | 44 | NA | 2.19 | 0.19 | 0.19 | 1.55 | 0.71 | 0.19 | 0.54 | 0.65 |
|  | Extreme Gradient Boosting (XgBoost) | All | 46 | 0.58 | 2.65 | 0.22 | 0.21 | 1.43 | 0.54 | 0.23 | 0.74 | 0.49 |
| Instance-Based Learners | |  |  |  |  |  |  |  |  |  |  |  |
|  | k-Nearest Neighbor (kKNN) |  |  |  |  |  |  |  |  |  |  |  |
|  |  | All | 38 | 0.60 | 2.22 | 0.20 | 0.19 | 1.47 | 0.66 | 0.20 | 0.61 | 0.58 |
|  |  | Mq | 8 | 0.50 | 1.12 | 0.03 | 0.03 | 1.05 | 0.94 | 0.02 | 0.56 | 0.47 |
|  |  | MqTurb | 54 | 0.65 | 2.67 | 0.24 | 0.24 | 1.71 | 0.64 | 0.23 | 0.58 | 0.66 |
|  |  | MPq | 35 | 0.61 | 2.25 | 0.14 | 0.15 | 1.86 | 0.83 | 0.20 | 0.31 | 0.83 |
|  |  | MPqW | 43 | 0.54 | 4.18 | 0.16 | 0.14 | 1.20 | 0.29 | 0.30 | 0.94 | 0.22 |
|  |  | MqW | 14 | 0.49 | 1.39 | 0.07 | 0.06 | 1.10 | 0.79 | 0.09 | 0.75 | 0.32 |
|  |  | Pq | 48 | 0.63 | 2.48 | 0.21 | 0.21 | 1.44 | 0.58 | 0.21 | 0.70 | 0.51 |
|  |  | PqW | 16 | 0.49 | 1.44 | 0.06 | 0.05 | 1.07 | 0.75 | 0.09 | 0.84 | 0.22 |
|  |  | S | 50 | 0.60 | 2.62 | 0.24 | 0.23 | 1.63 | 0.62 | 0.23 | 0.61 | 0.62 |
|  |  | W | 25 | 0.48 | 1.96 | 0.11 | 0.10 | 1.14 | 0.58 | 0.16 | 0.85 | 0.26 |
|  | Random kKNN (rKNN) | All | 23 | NA | 1.63 | 0.10 | 0.10 | 1.43 | 0.87 | 0.12 | 0.33 | 0.77 |
|  | Weighted kKNN (wKNN) | All | 46 | 0.58 | 2.62 | 0.23 | 0.23 | 1.75 | 0.67 | 0.23 | 0.54 | 0.69 |
| Penalized Regression | |  |  |  |  |  |  |  |  |  |  |  |
|  | Elastic Net | All | 33 | 0.59 | 2.06 | 0.17 | 0.17 | 1.53 | 0.74 | 0.18 | 0.50 | 0.67 |
|  | Lasso | All | 35 | 0.59 | 2.13 | 0.17 | 0.17 | 1.65 | 0.77 | 0.19 | 0.43 | 0.74 |
|  | Ridge |  |  |  |  |  |  |  |  |  |  |  |
|  |  | All | 66 | 0.66 | 5.40 | 0.31 | 0.29 | 1.55 | 0.29 | 0.36 | 0.88 | 0.44 |
|  |  | MqTurb | 43 | 0.63 | 2.26 | 0.20 | 0.19 | 1.42 | 0.63 | 0.20 | 0.66 | 0.53 |
|  |  | MPq | 54 | 0.64 | 2.98 | 0.23 | 0.22 | 1.42 | 0.48 | 0.26 | 0.79 | 0.45 |
|  |  | MPqW | 48 | 0.62 | 2.51 | 0.22 | 0.22 | 1.70 | 0.68 | 0.22 | 0.54 | 0.68 |
|  |  | MqW | 44 | 0.59 | 2.62 | 0.20 | 0.19 | 1.32 | 0.51 | 0.22 | 0.80 | 0.40 |
|  |  | Pq | 46 | 0.62 | 2.43 | 0.21 | 0.21 | 1.45 | 0.60 | 0.21 | 0.69 | 0.52 |
|  |  | PqW | 48 | 0.61 | 2.79 | 0.22 | 0.23 | 2.01 | 0.72 | 0.25 | 0.44 | 0.78 |
|  |  | S | 52 | 0.63 | 2.62 | 0.24 | 0.23 | 1.63 | 0.62 | 0.23 | 0.61 | 0.62 |
|  |  | W | 35 | 0.55 | 2.39 | 0.15 | 0.14 | 1.23 | 0.51 | 0.20 | 0.84 | 0.32 |
| Rule-Based Learners | |  |  |  |  |  |  |  |  |  |  |  |
|  | Repeated Incremental Pruning (JRip) | All | 45 | 0.61 | 2.48 | 0.21 | 0.21 | 1.76 | 0.71 | 0.22 | 0.49 | 0.72 |
|  | One Propositional Rule (OneR) | All | 21 | 0.54 | 2.05 | 0.07 | 0.08 | 1.89 | 0.92 | 0.18 | 0.15 | 0.92 |
|  | Partial Decision Lists (PART) |  |  |  |  |  |  |  |  |  |  |  |
|  |  | All | 23 | 0.53 | 1.98 | 0.12 | 0.13 | 1.68 | 0.85 | 0.17 | 0.30 | 0.82 |
|  |  | Mq | 9 | 0.53 | 1.45 | 0.07 | 0.07 | 1.32 | 0.91 | 0.09 | 0.29 | 0.78 |
|  |  | MqTurb | 16 | 0.53 | 1.64 | 0.11 | 0.11 | 1.40 | 0.85 | 0.13 | 0.38 | 0.73 |
|  |  | MPq | 46 | 0.59 | 2.47 | 0.22 | 0.22 | 1.53 | 0.62 | 0.22 | 0.64 | 0.58 |
|  |  | MPqW | 28 | 0.57 | 1.92 | 0.14 | 0.15 | 1.55 | 0.81 | 0.16 | 0.40 | 0.74 |
|  |  | MqW | 28 | 0.57 | 1.92 | 0.14 | 0.15 | 1.55 | 0.81 | 0.16 | 0.40 | 0.74 |
|  |  | Pq | 20 | 0.55 | 1.73 | 0.11 | 0.11 | 1.48 | 0.86 | 0.14 | 0.34 | 0.77 |
|  |  | PqW | 28 | 0.57 | 1.92 | 0.14 | 0.15 | 1.55 | 0.81 | 0.16 | 0.40 | 0.74 |
|  |  | S | 35 | 0.59 | 2.11 | 0.18 | 0.18 | 1.50 | 0.71 | 0.18 | 0.55 | 0.63 |
|  |  | W | 56 | 0.62 | 2.88 | 0.26 | 0.26 | 1.71 | 0.59 | 0.25 | 0.63 | 0.63 |
| Support Vector Machines | |  |  |  |  |  |  |  |  |  |  |  |
|  | Linear Kernel |  |  |  |  |  |  |  |  |  |  |  |
|  |  | All | 62 | 0.64 | 3.23 | 0.27 | 0.26 | 1.58 | 0.49 | 0.28 | 0.74 | 0.53 |
|  |  | Mq | 21 | 0.56 | 1.60 | 0.12 | 0.12 | 1.26 | 0.79 | 0.12 | 0.56 | 0.55 |
|  |  | MqTurb | 44 | 0.62 | 2.37 | 0.21 | 0.21 | 1.50 | 0.63 | 0.21 | 0.64 | 0.57 |
|  |  | MPq | 47 | 0.61 | 2.48 | 0.22 | 0.21 | 1.48 | 0.60 | 0.22 | 0.68 | 0.54 |
|  |  | MPqW | 40 | 0.60 | 2.38 | 0.18 | 0.19 | 1.81 | 0.76 | 0.21 | 0.41 | 0.77 |
|  |  | MqW | 51 | 0.61 | 3.11 | 0.21 | 0.20 | 1.34 | 0.43 | 0.27 | 0.84 | 0.38 |
|  |  | Pq | 36 | 0.60 | 2.13 | 0.18 | 0.18 | 1.40 | 0.65 | 0.19 | 0.65 | 0.53 |
|  |  | PqW | 36 | 0.59 | 2.46 | 0.15 | 0.16 | 2.02 | 0.82 | 0.23 | 0.30 | 0.85 |
|  |  | S | 51 | 0.62 | 2.62 | 0.24 | 0.23 | 1.63 | 0.62 | 0.23 | 0.61 | 0.62 |
|  |  | W | 27 | 0.56 | 1.88 | 0.15 | 0.15 | 1.29 | 0.68 | 0.15 | 0.68 | 0.48 |
|  | Polynomial Kernel | All | 67 | 0.63 | 5.68 | 0.34 | 0.32 | 1.64 | 0.29 | 0.38 | 0.86 | 0.48 |
|  | Radial Kernel | All | 37 | 0.49 | NA | 0.00 | 0.00 | NA | 1.00 | NA | 0.00 | 1.00 |
|  | Sigmoid Kernel | All | 67 | 0.64 | 4.42 | 0.32 | 0.31 | 1.68 | 0.38 | 0.34 | 0.80 | 0.52 |

^a^To assess the relative information gain associated with using different data types to build predictive models, two sets of analyses were performed. In the first set of analyses, each learner and the full set of factors listed in Table S1 were used to develop 23 predictive models per outcome. In the second set of analyses, the factors listed in Table S1 were divided into four groups: microbial data; physicochemical water quality and temperature data collected on site; weather data obtained from NEWA weather stations; and spatial data. Nested models were then built using different combinations of these 4 data types: Mq = microbial, MqTurb = microbial data and turbidity, Pq= Physicochemical water quality and air temperature collected on site; W= Weather from publicly-available databases; S = Spatial.

^b^ AUC stands for area under the curve. This is a measure of the accuracy of the model; specifically it is equal to the probability that the model will rank a randomly chosen positive sample higher than a randomly chosen negative sample. AUC ≤ 0.5 indicates the accuracy is equal to or worse than chance, while AUC=1 indicates a model with perfect accuracy.

^c^ DOR stands for diagnostic odds ratio. DOR < 1 indicates that a positive prediction is associated with a sample actually testing negative for the target pathogen, while DOR > 1 indicates that a positive prediction is associated with the sample actually testing positive for the target pathogen [see (16)].

^d^ A measure of how informed the model is about both positive and negative samples; value of 1 means that the pathogen status of all samples was correctly predicted, while a value of -1 implies the incorrect pathogen-status was predicted for all samples [see (17)].

^e^ Positive likelihood ratio; the larger the PLR for a given model, the more likely that a positive prediction actually corresponds to a sample that tested positive for the target pathogen. Values near 1 indicate a model with poor performance.

^f^ Negative likelihood ratio; the smaller the NLR for a given model, the more likely that a negative prediction actually corresponds to a sample that tested negative for the target pathogen. Values near 1 indicate a model with poor performance.

^g^ A measure of the trustworthiness of the model’s predictions; a value of 1 implies that all predictions are correct while a value of -1 implies all predictions are incorrect [see (17)].

^h^ Sensitivity or true positive rate.

^i^ Specificity or true negative rate.

Table S3: Performance measures for each model where *eaeA-stx* codetection was the outcome.

| **Learner** | | **Data Types^a^** | **Mean Rank** | **AUC^b^** | **DOR^c^** | **Informedness^d^** | **Kappa** | **PLR^e^** | **NLR^f^** | **Markedness^g^** | **Se^h^** | **Sp^i^** |
| --- | --- | --- | --- | --- | --- | --- | --- | --- | --- | --- | --- | --- |
| Bayesian Learners | |  |  |  |  |  |  |  |  |  |  |  |
|  | Naive Bayes | All | 32 | 0.61 | 2.42 | 0.20 | 0.20 | 1.37 | 0.57 | 0.19 | 0.74 | 0.46 |
| Baseline Models | |  |  |  |  |  |  |  |  |  |  |  |
|  | *E. coli* |  |  |  |  |  |  |  |  |  |  |  |
|  | 1,000 CFU/100-mL (EC.1000) | - | 40 | NA | 8.66 | 0.12 | 0.13 | 7.62 | 0.88 | 0.28 | 0.14 | 0.98 |
|  | 126 CFU/100-mL (EC.126) | - | 61 | NA | 6.76 | 0.44 | 0.44 | 2.66 | 0.39 | 0.39 | 0.71 | 0.73 |
|  | 235 CFU/100-mL (EC.235) | - | 49 | NA | 5.66 | 0.36 | 0.37 | 3.24 | 0.57 | 0.32 | 0.52 | 0.84 |
|  | 410 CFU/100-mL (EC.410) | - | 49 | NA | 27.83 | 0.32 | 0.34 | 18.82 | 0.68 | 0.38 | 0.34 | 0.98 |
|  | Total Coliforms |  |  |  |  |  |  |  |  |  |  |  |
|  | 1,000 CFU/100-mL (TC.1000) | - | 49 | NA | NA | 0.04 | 0.03 | 1.04 | 0.00 | 0.70 | 1.00 | 0.04 |
| Tree-based Learners | |  |  |  |  |  |  |  |  |  |  |  |
|  | Classification Tree (CART) | All | 29 | 0.56 | 2.35 | 0.11 | 0.13 | 1.14 | 0.49 | 0.20 | 0.90 | 0.21 |
|  | Conditional Inference Tree (CTree) |  |  |  |  |  |  |  |  |  |  |  |
|  |  | All | 29 | 0.56 | 2.35 | 0.11 | 0.13 | 1.14 | 0.49 | 0.20 | 0.90 | 0.21 |
|  |  | Mq | 63 | 0.71 | 6.85 | 0.42 | 0.42 | 2.08 | 0.30 | 0.42 | 0.82 | 0.61 |
|  |  | MqTurb | 63 | 0.71 | 6.85 | 0.42 | 0.42 | 2.08 | 0.30 | 0.42 | 0.82 | 0.61 |
|  |  | MPq | 63 | 0.71 | 6.85 | 0.42 | 0.42 | 2.08 | 0.30 | 0.42 | 0.82 | 0.61 |
|  |  | MPqW | 29 | 0.56 | 2.35 | 0.11 | 0.13 | 1.14 | 0.49 | 0.20 | 0.90 | 0.21 |
|  |  | MqW | 29 | 0.56 | 2.35 | 0.11 | 0.13 | 1.14 | 0.49 | 0.20 | 0.90 | 0.21 |
|  |  | Pq | 39 | 0.50 | NA | 0.00 | 0.00 | NA | 1.00 | NA | 0.00 | 1.00 |
|  |  | PqW | 29 | 0.56 | 2.35 | 0.11 | 0.13 | 1.14 | 0.49 | 0.20 | 0.90 | 0.21 |
|  |  | S | 38 | 0.50 | NA | 0.00 | 0.00 | NA | 1.00 | NA | 0.00 | 1.00 |
|  |  | W | 29 | 0.56 | 2.35 | 0.11 | 0.13 | 1.14 | 0.49 | 0.20 | 0.90 | 0.21 |
|  | Evolutionary Optimal Tree (EvTree) | All | 58 | 0.70 | 7.14 | 0.45 | 0.41 | 2.82 | 0.39 | 0.39 | 0.70 | 0.75 |
| Ensemble Learners | |  |  |  |  |  |  |  |  |  |  |  |
|  | Conditional Forest (condRF) | All | 49 | 0.68 | 3.76 | 0.31 | 0.30 | 1.68 | 0.45 | 0.29 | 0.75 | 0.55 |
|  | Node Harvest | All | 65 | 0.72 | 8.52 | 0.41 | 0.43 | 1.84 | 0.22 | 0.47 | 0.89 | 0.52 |
|  | Random Forest (RF) |  |  |  |  |  |  |  |  |  |  |  |
|  |  | All | 0.66 | 3.41 | 0.29 | 0.27 | 1.71 | 0.50 | 0.26 | 0.70 | 0.59 | 0.66 |
|  |  | Mq | 0.71 | 5.71 | 0.37 | 0.38 | 1.79 | 0.31 | 0.39 | 0.83 | 0.54 | 0.71 |
|  |  | MqTurb | 0.75 | 8.63 | 0.35 | 0.39 | 1.61 | 0.19 | 0.49 | 0.92 | 0.43 | 0.75 |
|  |  | MPq | 0.71 | 6.60 | 0.40 | 0.41 | 1.94 | 0.29 | 0.41 | 0.83 | 0.57 | 0.71 |
|  |  | MPqW | 0.68 | 3.75 | 0.32 | 0.28 | 1.99 | 0.53 | 0.28 | 0.64 | 0.68 | 0.68 |
|  |  | MqW | 0.66 | 3.93 | 0.31 | 0.31 | 1.70 | 0.43 | 0.30 | 0.76 | 0.55 | 0.66 |
|  |  | Pq | 0.69 | 3.96 | 0.33 | 0.30 | 1.88 | 0.47 | 0.30 | 0.70 | 0.63 | 0.69 |
|  |  | PqW | 0.60 | 2.67 | 0.21 | 0.22 | 1.39 | 0.52 | 0.22 | 0.77 | 0.45 | 0.60 |
|  |  | S | 0.58 | 2.21 | 0.19 | 0.16 | 1.59 | 0.72 | 0.16 | 0.51 | 0.68 | 0.58 |
|  |  | W | 0.56 | 2.65 | 0.20 | 0.21 | 1.34 | 0.51 | 0.22 | 0.79 | 0.41 | 0.56 |
|  | Regularized RF (regRF) | All | 52 | 0.68 | 4.10 | 0.32 | 0.32 | 1.72 | 0.42 | 0.31 | 0.77 | 0.55 |
|  | Random Ferns (rFern) | All | 45 | NA | 3.32 | 0.28 | 0.27 | 1.63 | 0.49 | 0.26 | 0.73 | 0.55 |
|  | Extreme Gradient Boosting (XgBoost) | All | 56 | 0.71 | 4.89 | 0.37 | 0.35 | 1.93 | 0.40 | 0.34 | 0.76 | 0.61 |
| Instance-Based Learners | |  |  |  |  |  |  |  |  |  |  |  |
|  | k-Nearest Neighbor (kKNN) |  |  |  |  |  |  |  |  |  |  |  |
|  |  | All | 32 | 0.56 | 4.86 | 0.20 | 0.14 | 3.81 | 0.78 | 0.25 | 0.27 | 0.93 |
|  |  | Mq | 61 | 0.73 | 7.73 | 0.30 | 0.35 | 1.48 | 0.19 | 0.47 | 0.93 | 0.38 |
|  |  | MqTurb | 42 | 0.61 | 3.03 | 0.22 | 0.23 | 1.36 | 0.45 | 0.25 | 0.82 | 0.39 |
|  |  | MPq | 28 | 0.52 | 2.38 | 0.06 | 0.08 | 1.07 | 0.45 | 0.20 | 0.95 | 0.11 |
|  |  | MPqW | 54 | 0.67 | 6.61 | 0.20 | 0.25 | 1.27 | 0.19 | 0.44 | 0.95 | 0.25 |
|  |  | MqW | 29 | 0.59 | 2.16 | 0.14 | 0.15 | 1.19 | 0.55 | 0.18 | 0.83 | 0.30 |
|  |  | Pq | 7 | 0.51 | 1.09 | 0.01 | 0.02 | 1.02 | 0.93 | 0.01 | 0.80 | 0.21 |
|  |  | PqW | 45 | 0.66 | 3.20 | 0.22 | 0.24 | 1.37 | 0.43 | 0.26 | 0.83 | 0.39 |
|  |  | S | 25 | 0.58 | 2.21 | 0.19 | 0.16 | 1.59 | 0.72 | 0.16 | 0.51 | 0.68 |
|  |  | W | 26 | 0.53 | 2.22 | 0.15 | 0.16 | 1.21 | 0.55 | 0.18 | 0.82 | 0.32 |
|  | Random kKNN (rKNN) | All | 20 | NA | 1.14 | 0.02 | 0.02 | 1.02 | 0.90 | 0.02 | 0.86 | 0.16 |
|  | Weighted kKNN (wKNN) | All | 38 | 0.60 | 4.82 | 0.28 | 0.21 | 3.26 | 0.68 | 0.28 | 0.41 | 0.88 |
| Penalized Regression | |  |  |  |  |  |  |  |  |  |  |  |
|  | Elastic Net | All | 22 | 0.58 | 1.89 | 0.12 | 0.13 | 1.18 | 0.62 | 0.14 | 0.80 | 0.32 |
|  | Lasso | All | 32 | 0.61 | 2.38 | 0.21 | 0.19 | 1.52 | 0.64 | 0.18 | 0.62 | 0.59 |
|  | Ridge |  |  |  |  |  |  |  |  |  |  |  |
|  |  | All | 26 | 0.59 | 2.14 | 0.19 | 0.17 | 1.44 | 0.67 | 0.16 | 0.62 | 0.57 |
|  |  | MqTurb | 41 | 0.66 | 3.31 | 0.29 | 0.26 | 1.81 | 0.55 | 0.25 | 0.65 | 0.64 |
|  |  | MPq | 46 | 0.69 | NA | 0.00 | 0.00 | NA | 1.00 | NA | 0.00 | 1.00 |
|  |  | MPqW | 44 | 0.62 | NA | 0.00 | 0.00 | NA | 1.00 | NA | 0.00 | 1.00 |
|  |  | MqW | 25 | 0.59 | 2.21 | 0.17 | 0.14 | 1.70 | 0.77 | 0.16 | 0.42 | 0.75 |
|  |  | Pq | 37 | 0.58 | 5.37 | 0.28 | 0.21 | 3.66 | 0.68 | 0.29 | 0.39 | 0.89 |
|  |  | PqW | 43 | 0.58 | NA | 0.04 | 0.05 | 1.04 | 0.00 | 0.70 | 1.00 | 0.04 |
|  |  | S | 10 | 0.49 | 1.14 | 0.02 | 0.02 | 1.02 | 0.90 | 0.02 | 0.86 | 0.16 |
|  |  | W | 41 | 0.53 | NA | 0.07 | 0.10 | 1.08 | 0.00 | 0.71 | 1.00 | 0.07 |
| Rule-Based Learners | |  |  |  |  |  |  |  |  |  |  |  |
|  | Repeated Incremental Pruning (JRip) | All | 29 | 0.56 | 2.35 | 0.11 | 0.13 | 1.14 | 0.49 | 0.20 | 0.90 | 0.21 |
|  | One Propositional Rule (OneR) | All | 40 | 0.56 | 3.15 | 0.12 | 0.15 | 1.15 | 0.37 | 0.27 | 0.93 | 0.20 |
|  | Partial Decision Lists (PART) |  |  |  |  |  |  |  |  |  |  |  |
|  |  | All | 31 | 0.49 | 2.44 | 0.13 | 0.15 | 1.17 | 0.48 | 0.20 | 0.88 | 0.25 |
|  |  | Mq | 60 | 0.67 | 8.02 | 0.33 | 0.37 | 1.56 | 0.19 | 0.48 | 0.92 | 0.41 |
|  |  | MqTurb | 51 | 0.60 | 7.39 | 0.16 | 0.21 | 1.20 | 0.16 | 0.46 | 0.97 | 0.20 |
|  |  | MPq | 16 | 0.48 | 1.71 | 0.09 | 0.10 | 1.13 | 0.66 | 0.13 | 0.82 | 0.27 |
|  |  | MPqW | 10 | 0.53 | 1.20 | 0.04 | 0.04 | 1.08 | 0.90 | 0.04 | 0.62 | 0.43 |
|  |  | MqW | 29 | 0.56 | 2.35 | 0.11 | 0.13 | 1.14 | 0.49 | 0.20 | 0.90 | 0.21 |
|  |  | Pq | 17 | 0.51 | 1.57 | 0.05 | 0.06 | 1.05 | 0.67 | 0.10 | 0.90 | 0.14 |
|  |  | PqW | 10 | 0.53 | 1.20 | 0.04 | 0.04 | 1.08 | 0.90 | 0.04 | 0.62 | 0.43 |
|  |  | S | 16 | 0.54 | 1.79 | 0.11 | 0.08 | 1.55 | 0.87 | 0.12 | 0.30 | 0.80 |
|  |  | W | 29 | 0.56 | 2.35 | 0.11 | 0.13 | 1.14 | 0.49 | 0.20 | 0.90 | 0.21 |
| Support Vector Machines | |  |  |  |  |  |  |  |  |  |  |  |
|  | Linear Kernel |  |  |  |  |  |  |  |  |  |  |  |
|  |  | All | 42 | 0.66 | 3.00 | 0.24 | 0.24 | 1.45 | 0.48 | 0.24 | 0.78 | 0.46 |
|  |  | Mq | 35 | 0.21 | NA | 0.00 | 0.00 | 1.00 | NA | NA | 1.00 | 0.00 |
|  |  | MqTurb | 14 | 0.45 | 2.09 | 0.04 | 0.02 | 2.02 | 0.96 | 0.14 | 0.07 | 0.96 |
|  |  | MPq | 42 | 0.56 | NA | 0.00 | 0.00 | NA | 1.00 | NA | 0.00 | 1.00 |
|  |  | MPqW | 29 | 0.61 | 2.25 | 0.18 | 0.18 | 1.33 | 0.59 | 0.18 | 0.74 | 0.45 |
|  |  | MqW | 43 | 0.64 | 3.03 | 0.22 | 0.23 | 1.36 | 0.45 | 0.25 | 0.82 | 0.39 |
|  |  | Pq | 39 | 0.46 | NA | 0.00 | 0.00 | NA | 1.00 | NA | 0.00 | 1.00 |
|  |  | PqW | 34 | 0.58 | 2.46 | 0.17 | 0.18 | 1.27 | 0.52 | 0.21 | 0.82 | 0.36 |
|  |  | S | 11 | 0.54 | 1.14 | 0.02 | 0.02 | 1.02 | 0.90 | 0.02 | 0.86 | 0.16 |
|  |  | W | 26 | 0.56 | 2.21 | 0.17 | 0.17 | 1.29 | 0.58 | 0.17 | 0.76 | 0.41 |
|  | Polynomial Kernel | All | 52 | 0.67 | 4.82 | 0.37 | 0.35 | 2.04 | 0.42 | 0.33 | 0.73 | 0.64 |
|  | Radial Kernel | All | 53 | 0.70 | 6.31 | 0.42 | 0.37 | 2.83 | 0.45 | 0.36 | 0.66 | 0.77 |
|  | Sigmoid Kernel | All | 41 | 0.64 | 2.87 | 0.21 | 0.22 | 1.34 | 0.47 | 0.24 | 0.82 | 0.39 |

^a^To assess the relative information gain associated with using different data types to build predictive models, two sets of analyses were performed. In the first set of analyses, each learner and the full set of factors listed in Table S1 were used to develop 23 predictive models per outcome. In the second set of analyses, the factors listed in Table S1 were divided into four groups: microbial data; physicochemical water quality and temperature data collected on site; weather data obtained from NEWA weather stations; and spatial data. Nested models were then built using different combinations of these 4 data types: Mq = microbial, MqTurb = microbial data and turbidity, Pq= Physicochemical water quality and air temperature collected on site; W= Weather from publicly-available databases; S = Spatial.

^b^ AUC stands for area under the curve. This is a measure of the accuracy of the model; specifically it is equal to the probability that the model will rank a randomly chosen positive sample higher than a randomly chosen negative sample. AUC ≤ 0.5 indicates the accuracy is equal to or worse than chance, while AUC=1 indicates a model with perfect accuracy.

^c^ DOR stands for diagnostic odds ratio. DOR < 1 indicates that a positive prediction is associated with a sample actually testing negative for the target pathogen, while DOR > 1 indicates that a positive prediction is associated with the sample actually testing positive for the target pathogen [see (16)].

^d^ A measure of how informed the model is about both positive and negative samples; value of 1 means that the pathogen status of all samples was correctly predicted, while a value of -1 implies the incorrect pathogen-status was predicted for all samples [see (17)].

^e^ Positive likelihood ratio; the larger the PLR for a given model, the more likely that a positive prediction actually corresponds to a sample that tested positive for the target pathogen. Values near 1 indicate a model with poor performance.

^f^ Negative likelihood ratio; the smaller the NLR for a given model, the more likely that a negative prediction actually corresponds to a sample that tested negative for the target pathogen. Values near 1 indicate a model with poor performance.

^g^ A measure of the trustworthiness of the model’s predictions; a value of 1 implies that all predictions are correct while a value of -1 implies all predictions are incorrect [see (17)].

^h^ Sensitivity or true positive rate.

^i^ Specificity or true negative rate.

1. Bridge Inventory Manual. Albany, NU. https://www.dot.ny.gov/divisions/engineering/structures/repository/manuals/inventory/2006_nysdot_inventory_manual_r.pdf.

2. Culvert Inventory and Inspection Manual. https://www.dot.ny.gov/divisions/operating/oom/transportation-maintenance/repository/CulvertInventoryInspectionManual.pdf.

3. NYS Large Culverts. https://gis.ny.gov/gisdata/inventories/details.cfm?DSID=1255.

4. Dams. https://gis.ny.gov/gisdata/inventories/details.cfm?DSID=1255

5. USGS National Transportation Dataset. https://gis.ny.gov/gisdata/inventories/details.cfm?DSID=1255.

6. Outfall and system mapping for illicit discharge detection and elimination (IDDE) in NY. Albany, NY. ftp://ftp.dec.state.ny.us/dow/stormdocuments/ms4/illicit_discharge_detection_and_elimination/illicit_discharge_detection_and_elimination_assistance/guidance/IDDE NYS Mapping Doc FINAL 06 12 05 for FTP site.pdf.

7. Guidance on outfall mapping: What is an outfall, and what should be mapped?. Albany, NY. http://www.dec.state.ny.us/website/dow/MS4crit.pdf.

8. Methodology for the identification and survey of stormwater outfalls within designated MS4 locations for New York State DOT. Albany, NY. https://www.dot.ny.gov/divisions/engineering/environmental-analysis/repository/OutfallMethodology.pdf.

9. State Pollutant Discharge Elimination System. https://gis.ny.gov/gisdata/inventories/details.cfm?DSID=1010.

10. National Hydrography Database. https://www.usgs.gov/core-science-systems/ngp/national-hydrography.

11. Septic Systems, New York State, 2011 - CUGIR. https://cugir.library.cornell.edu/catalog/cugir-008164.

12. Solid Waste Management Facilities | Open Data NY. https://data.ny.gov/Energy-Environment/Solid-Waste-Management-Facilities/2fni-raj8/data.

13. NLCD 2016 Land Cover (CONUS). Sioux Falls, SD. https://www.mrlc.gov.

14. NLCD 2016 Percent Developed Imperviousness. Sioux Falls, SD. https://www.mrlc.gov.

15. Center for Watershed Protection. Unified Stream Assessment: A User’s Manual. Ellicott City, MD. <https://owl.cwp.org/mdocs-posts/urban-subwatershed-restoration-manual-series-manual-10/>

16. **Weller D**, **Brassill N**, **Rock C**, **Ivanek R**, **Mudrak E**, **Roof S**, **Ganda E**, **Wiedmann M**. 2020. Complex Interactions Between Weather, and Microbial and Physicochemical Water Quality Impact the Likelihood of Detecting Foodborne Pathogens in Agricultural Water. Front Microbiol **11**.

17. **Powers D**. 2007. From Precision, Recall and F-Factor to ROC, Informedness, Markedness & Correlation.
